# Supplementary material for: HDAC5 enhances IRF3 activation and is targeted for degradation by protein C6 from orthopoxviruses including Monkeypox virus and Variola virus
Source: Cell Rep. 2024 Mar 10;43(3):113788. doi: 10.1016/j.celrep.2024.113788 (PMC11650635; doi:10.1016/j.celrep.2024.113788)
Supplement: Document S1. Figures S1‒S5 [file mmc1.pdf]

Cell Reports, Volume *43*

Supplemental information

HDAC5 enhances IRF3 activation and is targeted for degradation by protein C6 from orthopoxviruses including *Monkeypox virus* and *Variola virus*

Yongxu Lu (陆泳旭), Yiqi Zhao (赵奕祺), Chen Gao (高晨), Shreehari Suresh, Jinghao Men (门靖浩), Amelia Sawyers, and Geoffrey L. Smith

S 1

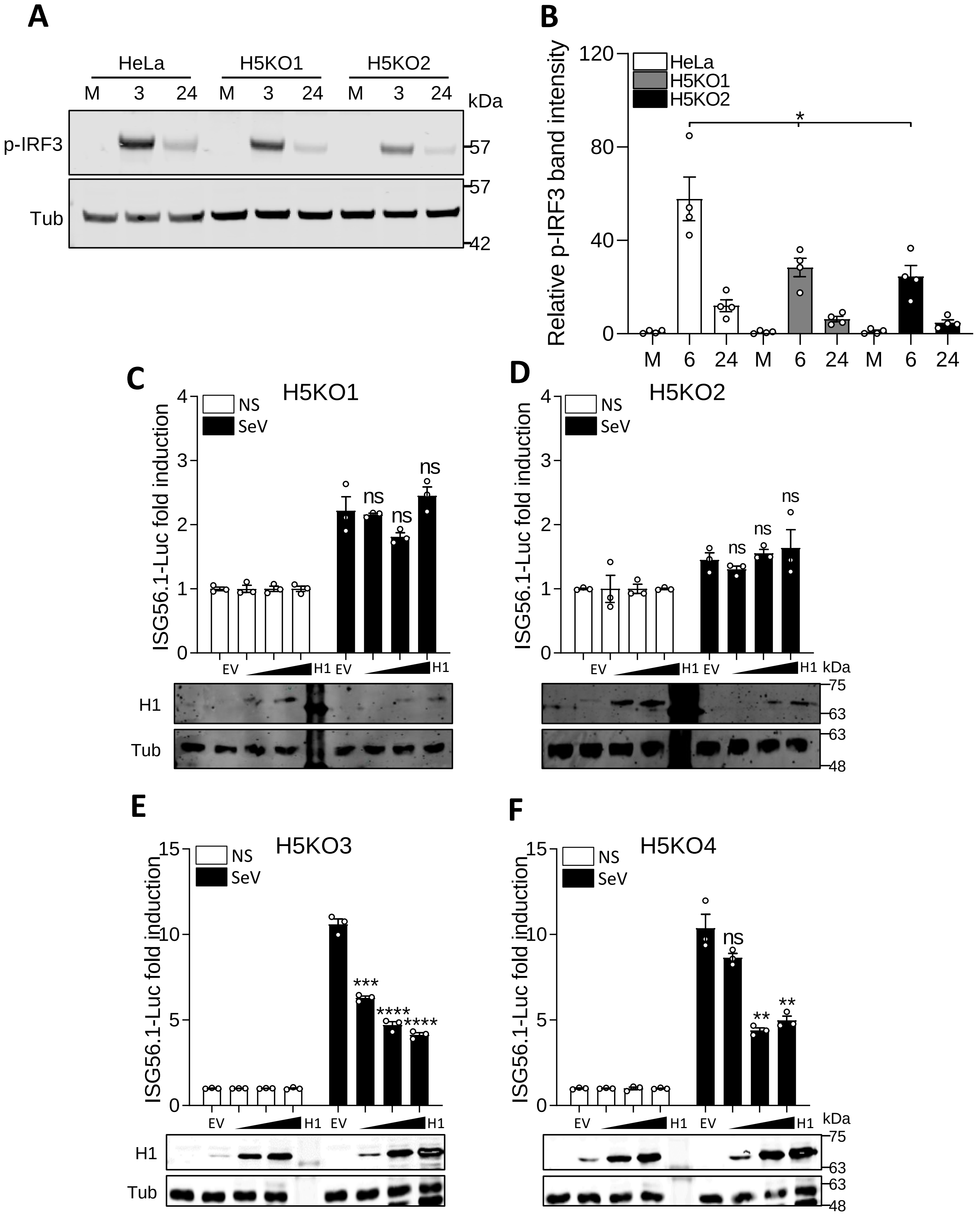

A
HeLa
H5KO1
H5KO2
M 3 24 M 3 24 M 3 24
kDa
p-IRF3
Tub
57
57
42
B
Relative p-IRF3 band intensity
HeLa
H5KO1
H5KO2
*
M 6 24 M 6 24 M 6 24
C
H5KO1
NS
SeV
ISG56.1-Luc fold induction
ns
EV H1 EV H1
H1
Tub
D
H5KO2
NS
SeV
ISG56.1-Luc fold induction
ns
EV H1 EV H1 kDa
H1
Tub
75
63
63
48
E
H5KO3
NS
SeV
ISG56.1-Luc fold induction
***
****
EV H1 EV H1
H1
Tub
F
H5KO4
NS
SeV
ISG56.1-Luc fold induction
ns
**
EV H1 EV H1 kDa
75
63
63
48

Fig. S1. IRF3 activation is attenuated in HDAC5$^{-/-}$ cells and cannot be rescued by HDAC1 or 4 over-expression. (Related to Fig. 1)

(A). Immunoblot showing p-IRF3 levels in parental HeLa and two HDAC5$^{-/-}$ cells. HeLa, H5KO1 and H5KO2 were infected with SeV, collected at different times p.i., and analysed by immunoblotting for levels of phosphorylated IRF3 (p-IRF3) and α-tubulin.

(B). The p-IRF3 level in (A) was calculated relative to α-tubulin (Tub) ± s.e.m. from 4 independent experiments. Data was analysed using one-way Welch's ANOVA test.

(C-F). Expression of HDAC1 does not rescue enhanced IRF3 pathway activation in HDAC5$^{-/-}$ cells. HDAC5$^{-/-}$ cells derived from HeLa (H5KO1 and H5KO2) or HEK293T (H5KO3 and H5KO4) cells were transfected with ISG56.1-Luc, TK-*Renilla* and increasing doses of plasmids expressing FLAG-tagged HDAC1 overnight. Transfected cells were then infected with SeV overnight to activate IRF3 pathway. The cells were then collected and firefly luciferase was measured and normalised to renilla luciferase control. The fold induction was calculated relative to unstimulated controls. Each condition was performed in triplicate. For each reporter gene assay, protein extracts were prepared and analysed by immunoblotting for FLAG-tagged HDAC1 and α-tubulin. The positions of molecular mass markers in kDa are indicated on the right.

Data are presented as mean ± s.e.m., n = 3 independent experiments. ns = not significant, *p < 0.05, **p < 0.01, ***p < 0.001, ****p < 0.0001.

S 2

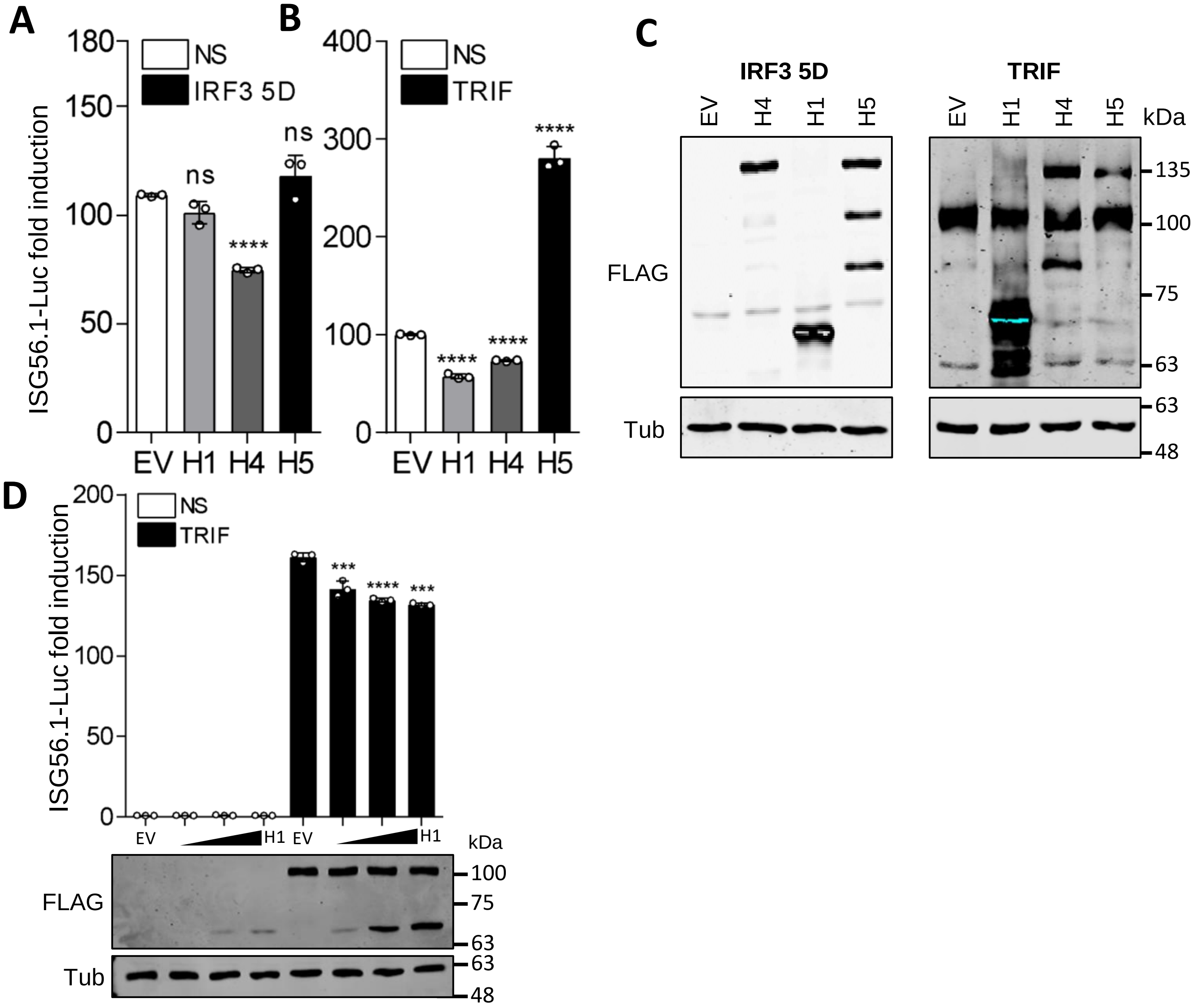

Fig. S2. HDAC5 enhances TRIF-mediated IRF3 activation. (Related to Fig. 3)

(A). HDAC5 does not enhance phospho-IRF-3–mediated IRF3 pathway activation. HEK293T cells seeded in 96-well plates were co-transfected with 100 ng ISG56.1-Luc, 10 ng TK-*Renilla*, 100 ng FLAG-tagged HDAC1, HDAC4, HDAC5 or empty vector (EV), and 5 ng of a mutant IRF3 (IRF3-5D). At 18 h post transfection, the cell lysates were collected and the firefly luciferase activity was measured and normalised to renilla luciferase as described in Fig. 1. Protein extracts were also used for immunoblotting as shown in (C).

(B). HDAC5, but not HDAC1 or HDAC4, enhances TRIF-mediated IRF3 activation in HEK293T cells. As in (A), except that 20 ng of TAP-tagged TRIFΔRIP was co-transfected to activate the IRF3 pathway for18 h. Cell lysates were then prepared and used to measure luciferase activity and for immunoblotting for expression of the FLAG-tagged proteins as shown in (C).

(C). Immunoblots for FLAG-tagged proteins and α-tubulin in cell lysates from (A) and (B).

(D). Expression of HDAC1 does not rescue TRIF-mediated IRF3 pathway activation in HDAC5$^{-/-}$ cells. H5KO3 cells were transfected with ISG56.1-Luc, TK-*Renilla*, TAP-TRIFΔRIP and HDAC1-FLAG expressing plasmids overnight. The HDAC1-FLAG expressing plasmid was transfected at 0, 10, 50, or 100 ng per well. The relative ISG56.1-Luc reporter expression was analysed as in Fig. 1. Bottom panels show immunoblots for HDAC1-FLAG, TAP-TRIFΔRIP and α-tubulin. In the immunoblots shown in (C) and (D), the positions of molecular mass markers in kDa are shown in the right.

Data are presented as mean ± s.e.m., n ≥ 3 independent experiments. ns = not significant, ***p < 0.001, ****p < 0.0001.

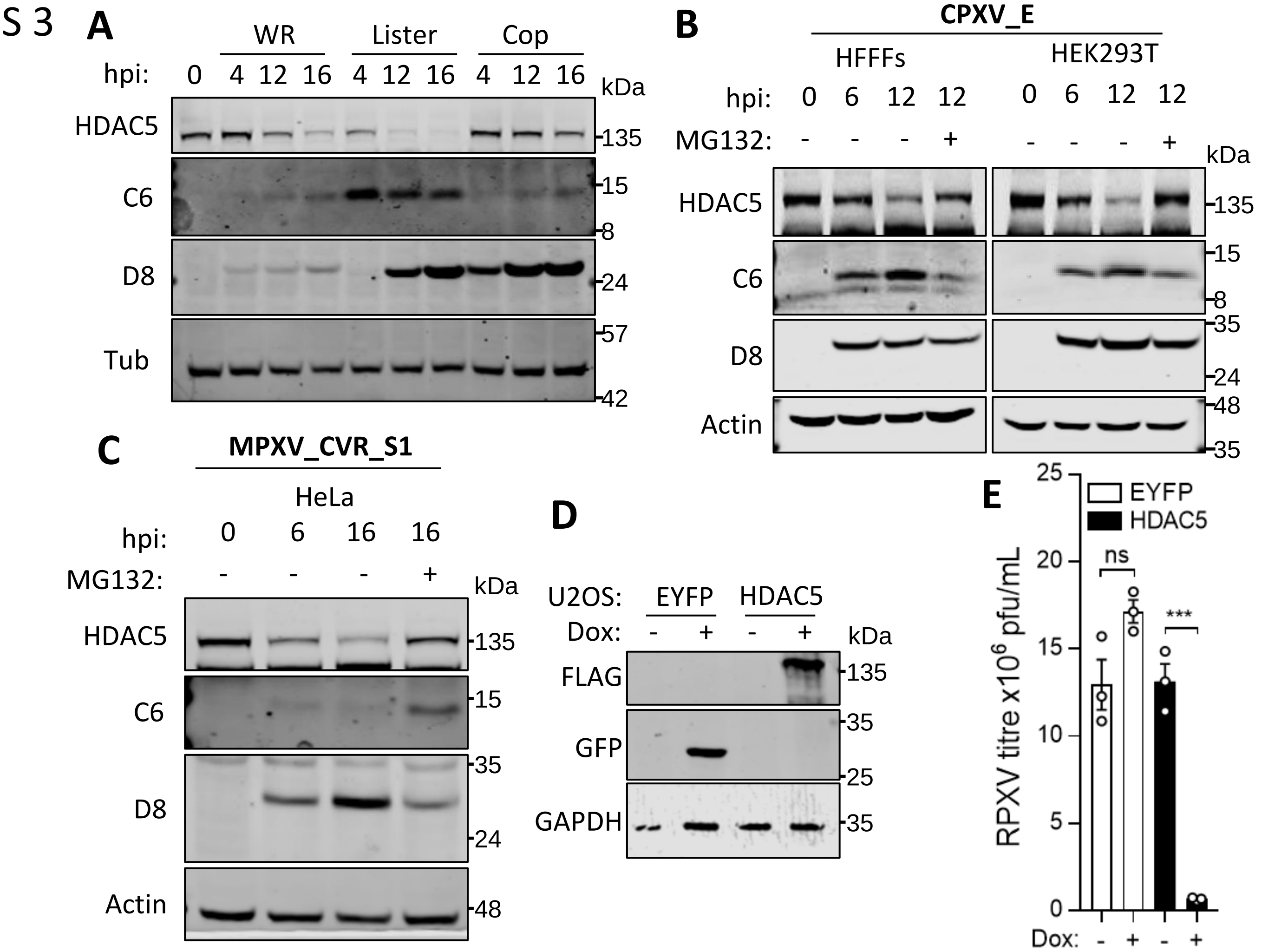

Fig. S3. HDAC5 restricts RPXV replication and is targeted for degradation by CPXV_E, MPXV_CVR_S1 and several VACV strains. (Related to Fig. 4)

(A). VACV strains WR, Copenhagen and Lister induce HDAC5 degradation during infection.

HFFFs were infected with VACV strains WR, Lister or Copenhagen (Cop) at 5 pfu per cell for 2 h and then the inoculum was replaced with fresh medium and incubated for the indicated times. Cell lysates were prepared and analysed by immunoblotting with the indicated antibodies.

(B). CPXV-E induces HDAC5 degradation during infection. As in (Fig. 4A), HFFFs or HEK293T cells were infected with CPXV-E at 5 pfu per cell for 2 h and then the inoculum was replaced with fresh medium supplemented with 10 µM MG132 (+) or equal volume of the carrier DMSO (-) and incubated for the indicated times. Cell lysates were prepared and analysed by immunoblotting with the indicated antibodies.

(C). As in (Fig. 4D). HeLa cells were infected with MPXV_CVR_S1 at 5 pfu per cell for 2 h and then the inoculum was replaced with fresh medium supplemented with 10 µM MG132 (+) or equal volume of the carrier DMSO (-) and incubated for the indicated times. Cell lysates were prepared and analysed by immunoblotting with the indicated antibodies.

(D). U2OS cells inducibly-expressing EYFP or HDAC5-FLAG. U2OS.TetR.EYFP or U2OS.TetR.HDAC5-FLAG cells were mock-induced (-) or induced with 100 ng/mL dox (+) for 18 h, and then cell lysates were prepared and analysed by immunoblotting. In (A-D), the positions of molecular mass markers are indicated in kDa on the right.

(E). Expression of HDAC5 in U2OS cells restricts RPXV replication. As in Fig. 4G. U2OS.TetR.EYFP or U2OS.TetR.HDAC5-FLAG cells were mock-induced (-) or induced with 100 ng/mL dox for 18 h (+) and then infected with RPXV at 0.01 pfu per cell for 2 d. The infectious virus present in the culture supernatant and infected cells was determined by plaque assay on BSC-1 cells.

Data are presented as mean ± s.e.m., n ≥ 3 independent experiments. ns = not significant, ***$p < 0.001$.

S 4

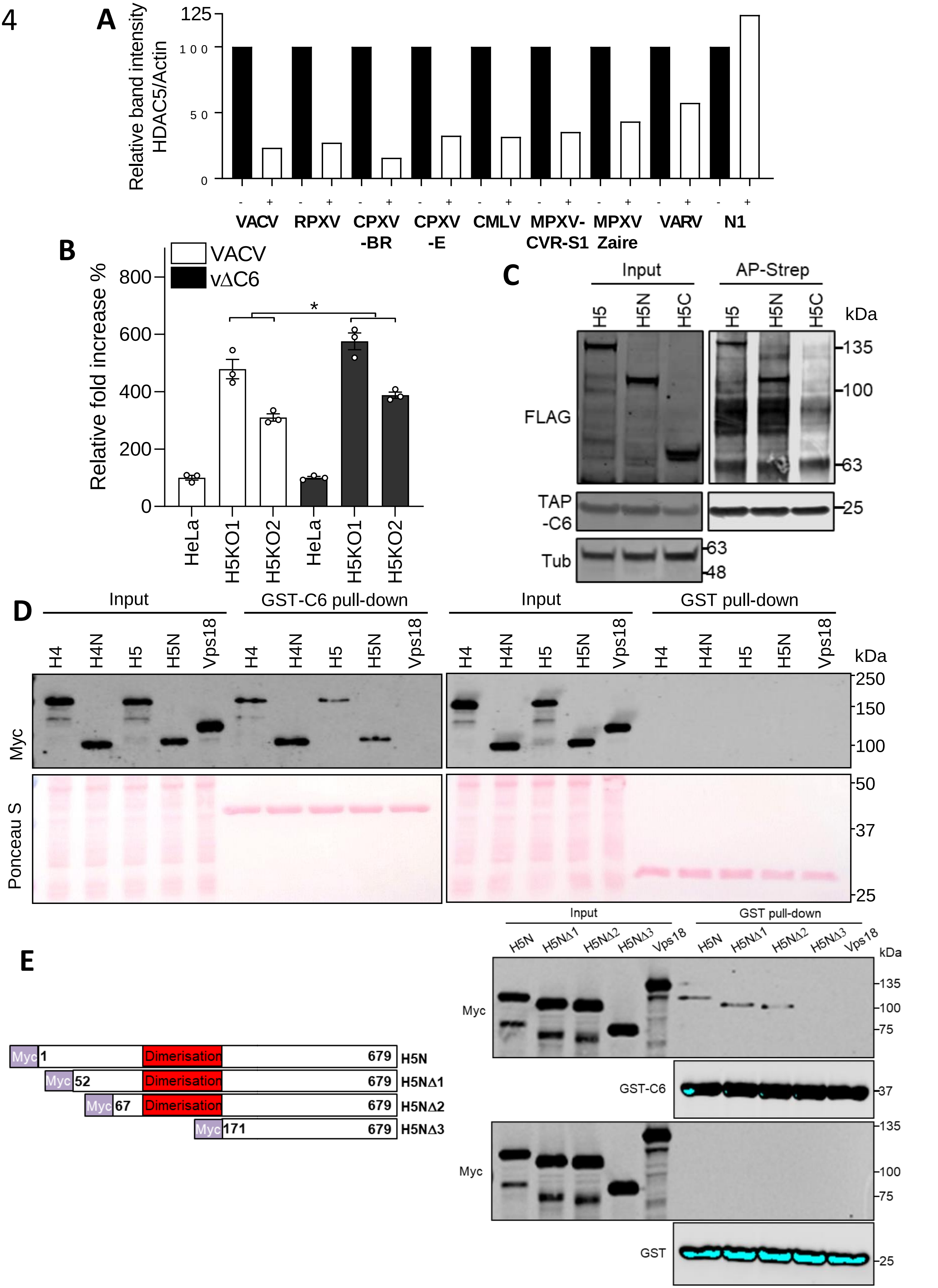
A
Relative band intensity HDAC5/Actin
125
100
50
0
- + - + - + - + - + - + - + - + - +
VACV
RPXV
CPXV -BR
CPXV -E
CMLV
MPXV- CVR-S1
MPXV Zaire
VARV
N1
B
VACV
vΔC6
Relative fold increase %
800
600
400
200
0
*
HeLa
H5KO1
H5KO2
HeLa
H5KO1
H5KO2
C
Input
AP-Strep
H5
H5N
H5C
H5
H5N
H5C
kDa
FLAG
TAP -C6
Tub
135
100
63
25
63
48
D
Input
GST-C6 pull-down
Input
GST pull-down
H4
H4N
H5
H5N
Vps18
kDa
Myc
Ponceau S
250
150
100
50
37
25
E
Myc
1
Dimerisation
679
H5N
52
H5NΔ1
67
H5NΔ2
171
H5NΔ3
Input
GST pull-down
H5N
H5NΔ1
H5NΔ2
H5NΔ3
Vps18
kDa
Myc
GST-C6
GST
135
100
75
37
25

Fig. S4. C6 interacts directly with the N-terminal region of HDAC4 and HDAC5 leading to their degradation. (Related to Fig. 5).

(A). Relative HDAC5 abundance in cells expressing (+) or not expressing (-) C6 proteins from different orthopoxviruses. The graph shows the band intensity of HDAC5 from Fig. 5D normalised to actin and comparing the mock-induced (-, 100%) to dox induced (+) samples.

(B). Deletion of C6 enhances the difference in viral yield between HeLa and HDAC5$^{-/-}$ cells. Parental HeLa, H5KO1 and H5KO2 cells were infected with vΔC6 or wt VACV at MOI=0.01. Two d p.i., the supernatant and infected cells were collected and infectious virus was titrated by plaque assay on BSC-1 cells. Data was analysed using two-way Welch's ANOVA test. n = 3 independent experiments. *$p < 0.05$

(C). C6 co-precipitates with the HDAC5 N-terminal region. HEK293T cells were co-transfected with plasmids encoding TAP-C6 and FLAG-tagged full length HDAC5, N-terminal HDAC5 (H5N, aa 1-679) or C-terminal HDAC5 (H5C, aa 680-1122). Cell lysates were prepared and TAP-C6 was AP by Strep-Tactin beads. Inputs and AP proteins were analysed as described in (Fig. 5).

(D) HDAC5 and HDAC4 bind directly to C6. The indicated myc-tagged HDAC4 (H4), N-terminal HDAC4 (H4N, aa 1-650), HDAC5 (H5), H5N or Vsp18 were expressed *in vitro* using the wheat germ cell-free transcription and translation system. The *in vitro* expressed proteins were AP with glutathione beads bound to glutathione S-transferase (GST)-tagged C6 (middle panel) or GST (bottom panel). The AP proteins were analysed by immunoblotting using anti-myc antibody and the GST-C6 and GST were stained with ponceau S.

(E). More detailed mapping of the HDAC5 region required for binding C6. Same as in (D) The indicated myc-tagged HDAC5 mutants (top panel) or Vsp18 were expressed *in vitro* and AP with glutathione beads bound to glutathione S-transferase (GST)-tagged C6 (middle panel) or GST (bottom panel). The AP proteins were analysed by immunoblotting using anti-myc or anti-GST antibodies. In (C-E) the positions of molecular mass markers are shown in kDa on the right.

S 5

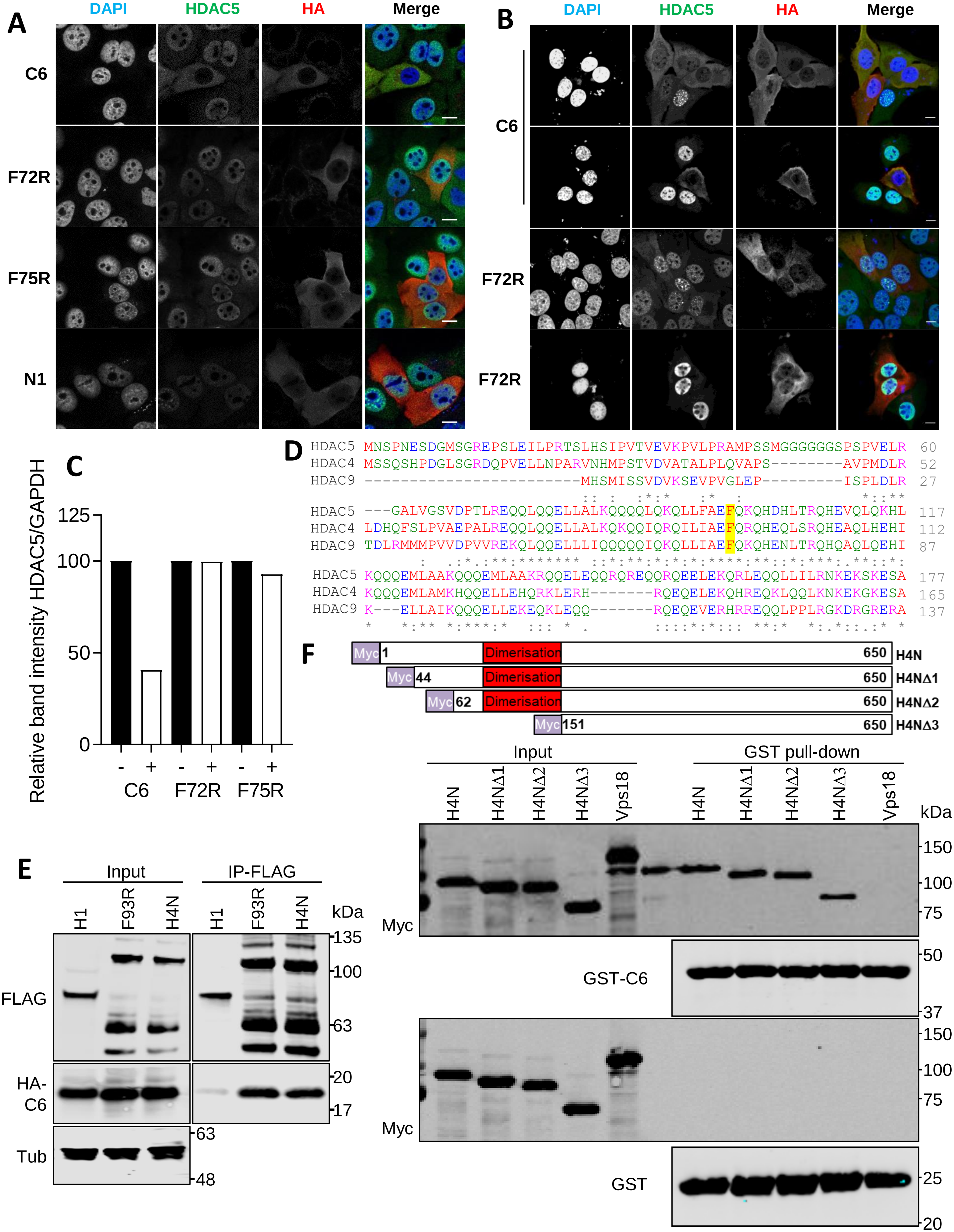
A
B
C
D
E
F
DAPI
HDAC5
HA
Merge
C6
F72R
F75R
N1
Relative band intensity HDAC5/GAPDH
125
100
50
0
- + - + - +
C6
F72R
F75R
HDAC5 MNSPNESDGMSGREPSLEILPRTSLHSIPVTVEVKPVLPRAMPSSMGGGGGGSPSPVELR 60
HDAC4 MSSQSHPDGLSGRDQPVELLNPARVNHMPSTVDVATALPLQVAPS---------AVPMDLR 52
HDAC9 ------------------------MHSMISSVDVKSEVPVGLEP----------ISPLDLR 27
HDAC5 ---GALVGSVDPTLREQQLQQELLALKQQQQLQKQLLFAEFQKQHDHLTRQHEVQLQKHL 117
HDAC4 LDHQFSLPVAEPALREQQLQQELLALKQKQQIQRQILIAEFQRQHEQLSRQHEAQLHEHI 112
HDAC9 TDLRMMMPVVDPVVREKQLQQELLLIQQQQQIQKQLLIAEFQKQHENLTRQHQAQLQEHI 87
HDAC5 KQQQEMLAAKQQQEMLAAKRQQELEQQRQREQQRQEELEKQRLEQQLLILRNKEKSKESA 177
HDAC4 KQQQEMLAMKHQQELLEHQRKLERH--------RQEQELEKQHREQKLQQLKNKEKGKESA 165
HDAC9 K---ELLAIKQQQELLEKEQKLEQQ--------RQEQEVERHRREQQLPPLRGKDRGRERA 137
Input
IP-FLAG
H1
F93R
H4N
kDa
135
100
63
20
17
48
FLAG
HA-C6
Tub
Myc
Dimerisation
1
44
62
151
650
H4N
H4NΔ1
H4NΔ2
H4NΔ3
GST pull-down
Vps18
150
75
50
37
25
GST-C6
GST

Fig. S5. C6 induces HDAC5 re-localisation and interacts directly with multiple binding sites in the HDAC4 N-terminal region (Related to Figs. 6 & 7).

(A). HDAC5 re-localisation to the cytoplasm is induced by C6, but not C6 mutants F72R or F75R. U2OS.HDAC5 cells were transfected with 0.5 µg of plasmids expressing HA-tagged N1, C6 or C6 mutants F72R or F75R for 24 h. Cells were induced with 100 ng/mL dox to express HDAC5-FLAG overnight. Cells were then fixed and immunostained with anti-FLAG and anti-HA antibodies, and DNA was stained with DAPI. Cell images were obtained via confocal microscopy. Scale bar, 20 µm.

(B). Independent immunofluorescence images showing that C6, but not C6 mutants F72R and F75R, induced HDAC5 re-localisation. Scale bar, 20 µm.

(C). Relative HDAC5 abundance in cells expressing (+) or not expressing (-) C6 or C6 mutants F72R and F75R. The graph shows the band intensity of HDAC5 from Fig. 6G normalised to GAPDH and comparing the mock-induced (-, 100%) to dox induced (+) samples.

(D). Amino acid sequence alignment of HDAC5, HDAC4 and HDAC9. The position of HDAC5 F98 and the corresponding aa in HDAC4 and HDAC9 are highlighted in yellow.

(E). HDAC4 F93 is not essential for HDAC4 and C6 co-precipitation. TAP-tagged C6 and FLAG-tagged HDAC1, HDAC4-N or HDAC4-N with a F93R mutation were co-expressed in HEK293T cells by transfection and were precipitated with anti-FLAG antibody. Input (left) and purified proteins (right) were analysed by immunoblotting with the indicated antibodies.

(F). Mapping of HDAC4 domain(s) required for binding C6. As in Fig. S4C, except that myc-tagged HDAC4 mutants (top panel) were expressed *in vitro* using the wheat-germ cell free transcription-translation system and were AP with either GST-C6 (middle panel) or GST (bottom panel). In C & D, the positions of molecular mass markers are shown in kDa on the right.
