# Supplementary material for: HDAC5 enhances IRF3 activation and is targeted for degradation by protein C6 from orthopoxviruses including Monkeypox virus and Variola virus
Source: Cell Rep. 2024 Mar 10;43(3):113788. doi: 10.1016/j.celrep.2024.113788 (PMC11650635; doi:10.1016/j.celrep.2024.113788)
Supplement: Table S1. Oligonucleotide sequences, related to STAR Methods [file mmc2.pdf]

Table S1

Oligonucleotides		
HDAC5N_FWD TTATAAGCTTGCCACCATGAACTCTCCCAACGAGTCGGATGGGATGTCAGG	This paper	N/A
HDAC5N_REV GGCCTCTAGACTCGAGTTATTTATCATCATCATCTTTATAATCCCCTGTGGTGAAGAGGTGC	This paper	N/A
HDAC5C_FWD TTATAAGCTTGCCACCATGGGTGTGGTCTACGACACGTTC	This paper	N/A
HDAC5C_REV GGCCTCTAGACTCGAGTTATTTATCATCATCATCTTTATAATC	This paper	N/A
HDAC4N_FWD CTCAGGATCCGGGCCACCATGAGCTCCCAAAGCCATCC	This paper	N/A
HDAC4N_REV GGCGAATTCCGCTTATTTATCATCATCATCTTTATAATCCGCGGCCGCCGGCTTGGTGGGGGGCTC	This paper	N/A
HDAC4C_FWD AATTGGATCCGCCACCATGAGGTTCACGACAGGCCTCGTG	This paper	N/A
HDAC4C_REV AATTGAATTCCGCTTATTTATCATCATCATCTTTATAATCCCCC	This paper	N/A
HDAC5_H893F_FWD TACATCTCTCTGTTCCGCTAT	This paper	N/A
HDAC5_H893F_FWD2 CCCCTCTGTGCTCTACATCTCTCTGTTCCGCTATGACAACGGG	This paper	N/A
HDAC5_H893F_REV ATAGCGGAACAGAGAGATGTA	This paper	N/A
HDAC5_H893F_REV2 CCCGTTGTCATAGCGGAACAGAGAGATGTAGAGCACAGAGGGG	This paper	N/A
HDAC5N_FLAG to HA_FWD GCCGGATTATGCGTAACTCGAGTCTAGAGGGC	This paper	N/A
HDAC5N_FLAG to HA_REV ACATCATACGGATACCCTGTGGTGAAGAGGTG	This paper	N/A
Codon optimized C6_FWD CTAGCGTTTAAACTTAAGCTTGGTACCGGATCCATGTGG	This paper	N/A
Codon optimized C6_REV TTAAACGGGCCCTCTAGATTATCATCTGTCCACGTC	This paper	N/A
Codon optimized C6_F72R_FWD GCCAAGGACATCAACGCCATGAGCAGAGACGGCTTC	This paper	N/A
Codon optimized C6_F72R_REV GAAGCCGTCTCTGCTCATGGCGTTGATGTCCTTGGC	This paper	N/A

Codon optimized C6_F75R_FWD ATCAACGCCATGAGCTTCGACGGCAGAATCAGAAGC	This paper	N/A
Codon optimized C6_F75R_REV GCTTCTGATTCTGCCGTCGAAGCTCATGGCGTTGAT	This paper	N/A
HDAC5_F95R_FWD GCAGCTCCTGCGTGCTGAGTTCC	This paper	N/A
HDAC5_F95R_REV TTCTGCAGCTGCTGC	This paper	N/A
HDAC5_F98R_FWD GTTCGCTGAGCGTCAGAAACAGCATG	This paper	N/A
HDAC5_F98R_REV AGGAGCTGCTTCTGC	This paper	N/A
HDAC4_F93R_FWD CATCGCTGAGCGCCAGAGGCAGC	This paper	N/A
HDAC4_F93R_REV AGGATCTGCCTCTGGATC	This paper	N/A
HDAC4_F94R_FWD CATCGCTGAGCGCCAGAGGCAGC	This paper	N/A
HDAC4_F94R_REV AGGATCTGCCTCTGGATC	This paper	N/A
