# Supplementary material for: HDAC5 enhances IRF3 activation and is targeted for degradation by protein C6 from orthopoxviruses including Monkeypox virus and Variola virus
Source: Cell Rep. 2024 Mar 10;43(3):113788. doi: 10.1016/j.celrep.2024.113788 (PMC11650635; doi:10.1016/j.celrep.2024.113788)
Supplement: Document S2. Article plus supplemental information [file mmc5.pdf]

Cell Reports

Article

HDAC5 enhances IRF3 activation and is targeted for degradation by protein C6 from orthopoxviruses including *Monkeypox virus* and *Variola virus*

Graphical abstract

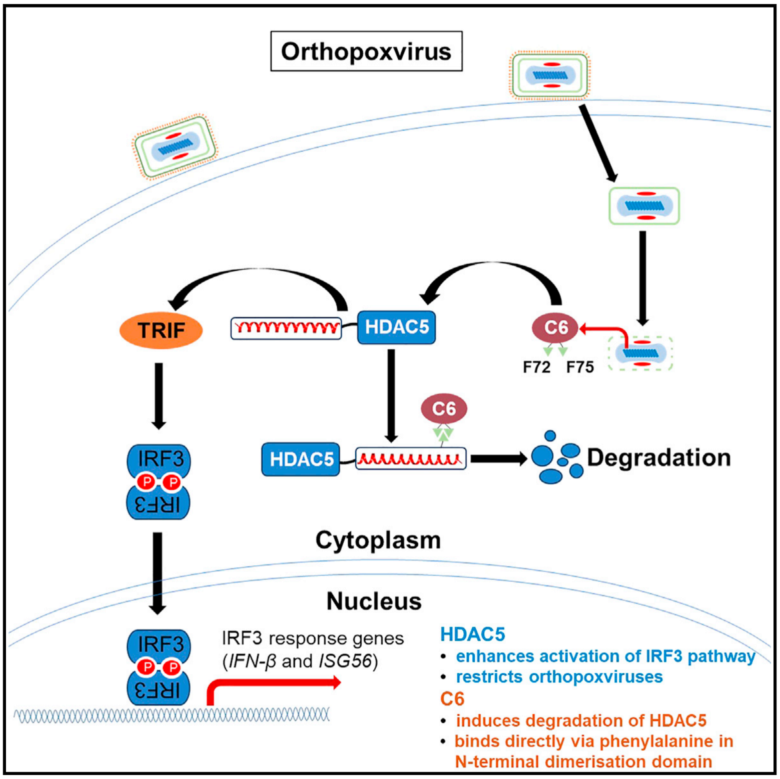

Highlights

- HDAC5 promotes TRIF-mediated IRF3 activation
- HDAC5 restricts multiple orthopoxviruses and is degraded during virus infection
- Orthopoxvirus protein C6 induces HDAC5 degradation
- Protein C6 binds directly to HDAC5 dimerization domain, thereby preventing dimerization

Authors

Yongxu Lu, Yiqi Zhao, Chen Gao, Shreehari Suresh, Jinghao Men, Amelia Sawyers, Geoffrey L. Smith

Correspondence

yongxu.lu@path.ox.ac.uk (Y.L.), geoffrey.smith@path.ox.ac.uk (G.L.S.)

In brief

HDAC5 enhances TRIF-mediated IRF3 activation and restricts several orthopoxviruses. Lu et al. show that the C6 protein of these orthopoxviruses induces degradation of HDAC5 and interacts directly with HDAC5 via the HDAC5 N-terminal dimerization domain.

Lu et al., 2024, Cell Reports *43*, 113788
March 26, 2024 © 2024 The Authors.
https://doi.org/10.1016/j.celrep.2024.113788

CellPress

CellPress
OPEN ACCESS

Article

HDAC5 enhances IRF3 activation and is targeted for degradation by protein C6 from orthopoxviruses including *Monkeypox virus* and *Variola virus*

Yongxu Lu (陆泳旭),[1,2,4,*] Yiqi Zhao (赵奕祺),[1,2,4] Chen Gao (高晨),[1,5] Shreehari Suresh,[1,6] Jinghao Men (门靖浩),[1,7] Amelia Sawyers,[1,8] and Geoffrey L. Smith[1,2,3,4,9,*]
[1]Department of Pathology, University of Cambridge, Tennis Court Road, Cambridge CB2 1QP, UK
[2]Sir William Dunn School of Pathology, University of Oxford, South Parks Road, Oxford OX1 3RE, UK
[3]The Pirbright Institute, Surrey, UK
[4]Chinese Academy of Medical Sciences-Oxford Institute, University of Oxford, Oxford, UK
[5]Present address: WaveBreak, Chemistry of Health, Lensfield Road, Cambridge CB2 1EW, UK
[6]Present address: Buckinghamshire NHS Healthcare Trust, Stoke Mandeville Hospital, Aylesbury HP21 8AL, UK
[7]Present address: Department of Biological Chemistry and Molecular Pharmacology, Harvard Medical School, Boston, MA 02115, USA
[8]Present address: Vanderbilt University School of Medicine, 1161 21st Ave. S, #D300, Nashville, TN, USA
[9]Lead contact
*Correspondence: yongxu.lu@path.ox.ac.uk (Y.L.), geoffrey.smith@path.ox.ac.uk (G.L.S.)
https://doi.org/10.1016/j.celrep.2024.113788

SUMMARY

Histone deacetylases (HDACs) regulate gene expression and innate immunity. Previously, we showed that HDAC5 is degraded during *Vaccinia virus* (VACV) infection and is a restriction factor for VACV and herpes simplex virus type 1. Here, we report that HDAC5 promotes interferon regulatory factor 3 (IRF3) activation downstream of Toll-IL-1 receptor (TIR) domain-containing adaptor molecule-1 or Sendai virus-mediated stimulation without requiring HDAC activity. Loss of HDAC5-mediated IRF3 activation is restored by re-introduction of HDAC5 but not HDAC1 or HDAC4. The antiviral activity of HDAC5 is antagonized by VACV protein C6 and orthologs from the orthopoxviruses cowpox, rabbitpox, camelpox, monkeypox, and variola. Infection by many of these viruses induces proteasomal degradation of HDAC5, and expression of C6 alone can induce HDAC5 degradation. Mechanistically, C6 binds to the dimerization domain of HDAC5 and prevents homodimerization and heterodimerization with HDAC4. Overall, this study describes HDAC5 as a positive regulator of IRF3 activation and provides mechanistic insight into how the poxviral protein C6 binds to HDAC5 to antagonize its function.

INTRODUCTION

The *Poxviridae* is a family of large double-stranded DNA (dsDNA) viruses that infect insects (*Entomopoxvirinae*) and chordates (*Chordopoxvirinae*).[1] Within the *Chordopoxvirinae*, the *Orthopoxvirus* genus is the most intensively studied and includes *Vaccinia virus* (VACV), *Cowpox virus* (CPXV), *Camelpox virus* (CMLV), *Monkeypox virus* (MPXV), and *Variola virus* (VARV), the cause of smallpox. Members of this genus are immunologically cross-reactive and cross-protective and, consequently, CPXV and VACV were effective vaccines against smallpox, leading to its eradication in 1979.[2] VACV is also the vaccine being used currently against MPXV. VACV is the prototypic poxvirus and has been used to study many features of virus replication and virus-host interactions. It has also been used widely as an expression vector,[3,4] as a candidate vaccine for other infectious diseases,[5–7] and as an oncolytic agent.[8,9]

VACV encodes many proteins that block the innate immune response to infection, and these function at multiple levels.[1,10] One strategy is to degrade host proteins that have antiviral activity and thereby escape host restriction and promote replication. A temporal proteomics analysis of VACV-infected human fibroblasts showed that 265 host proteins are downregulated more than 2-fold, some of which have been identified as restriction factors for VACV.[11–13] This study concerns one of these downregulated proteins, histone deacetylase 5 (HDAC5).

HDACs are a family of proteins that regulate cellular gene expression and innate immunity at the transcriptional level by epigenetic modification of chromatin[14,15] and at post-transcriptional levels.[11,16–19] HDAC3, HDAC4, HDAC5, and HDAC11 restrict infection of some viruses via regulation of IRF3, nuclear factor κB (NF-κB), or type I interferon (IFN) Janus kinase-signal transducers and activators of transcription (JAK-STAT) signaling pathways.[11,16,18,19] Dysregulation of HDACs is also involved in many diseases, such as neurodegenerative and metabolic disorders, cardiac hypertrophy, and cancer.[20–22] Human HDACs are classified into five subfamilies, class I, IIa, IIb, III and IV, based on phylogenetic comparison with yeast orthologs.[23] All HDACs

This is an open access article under the CC BY license (http://creativecommons.org/licenses/by/4.0/).

have a highly conserved C-terminal domain with HDAC enzymatic activity, but unlike the other subfamilies, class IIa HDACs have an additional N-terminal region that mediates homo- or heterodimerization with other class II HDACs and interaction with proteins 14-3-3 and MEF2.[24] Previously, the class II HDACs, HDAC4 and HDAC5, were found to be degraded during VACV infection, while the levels of other HDACs were unaltered.[12] The targeted degradation of HDAC4 is likely due to its ability to restrict VACV and herpes simplex virus type 1 (HSV-1) replication and its involvement in the recruitment of STAT2 to the IFN-stimulated response element (ISRE) promoter in response to type I IFN-induced signaling.[11] Notably, HDAC4, HDAC5, and TRIM5α are all targeted by VACV early protein C6 for proteasome-dependent degradation.[11–13]

HDAC5 has antiviral activity against several DNA viruses and is not only targeted by VACV protein C6[12] but also by HSV-1 protein ICP0[25] and Kaposi's sarcoma-associated herpesvirus (KSHV) viral IFN regulatory factor 3 (vIRF3) to regulate gene expression in lymphatic endothelial cells.[26] Additionally, HDAC5 represses EBNA2-activated Epstein-Barr virus (EBV) LMP1p and Cp promoters, suggesting that it may be antiviral against EBV.[27] In contrast, the enzymatic domain of HDAC5 increases both the stability and splicing of the hepatitis B virus (HBV) 3.5-kb RNA, therefore enhancing HBV biosynthesis.[28] Human cell lines lacking HDAC5 support enhanced replication of VACV and HSV-1, and re-introduction of HDAC5 into these *HDAC5*$^{-/-}$ cells reversed this phenotype.[12] Nonetheless, the mechanism by which HDAC5 restricts VACV infection is unclear.

In this study, we show that HDAC5 positively regulates the activation of the IRF3 pathway and is targeted for degradation by several orthopoxviruses, including VACV, CPXV, CMLV, and MPXV_CVR_S1. This degradation is induced by orthopoxvirus protein C6, which interacts directly with HDAC5 via the HDAC5 N-terminal region. Alphafold was used to predict the structure of C6 in complex with the N-terminal domain of HDAC5 and indicated that the interaction is mediated by a hydrophobic pocket formed by three phenylalanines, two of which are on C6, and the third one is on HDAC5. The latter is conserved in other type II HDACs and is needed for HDAC5 homodimerization or heterodimerization with HDAC4. Mutation of these phenylalanines abolished the interaction between C6 and HDAC5. Additionally, we demonstrate that C6 disrupts HDAC5 homodimerization and heterodimerization with HDAC4.

RESULTS

HDAC5 is a positive regulator of the IRF3 pathway

Previous work demonstrated that HDAC5 is a restriction factor for VACV and HSV-1, but how its antiviral activity is mediated was not determined. Given that VACV encodes many proteins to block innate immunity, and HDAC5 was targeted for degradation during VACV infection, we investigated whether HDAC5 has a role in innate immunity by screening the ability of HDAC5 to modulate activation of intracellular signaling pathways. We selected activation of the transcription factors IRF3 and NF-κB as well as those in the JAK-STAT pathways by stimulation with type I and type II IFN for analysis. Pathway activation was measured by reporter gene assays in parental HeLa cells and two *HDAC5*$^{-/-}$ cell lines (H5KO1 and H5KO2).[12] Cells were transfected with plasmids expressing firefly luciferase driven by the (ISRE-Luc), IFN-γ-activated sequence (interferon-gamma-activated site (GAS)-Luc), NF-κB (NF-κB-Luc), or ISG56 (ISG56.1-Luc) promoter, along with a transfection control plasmid expressing *Renilla* Luc (human thymidine kinase (TK) promoter-*Renilla*). Following cytokine stimulation or Sendai virus (SeV) infection, transfected cells were collected, and pathway activation was assessed by measurement of Luc activity. The result showed that loss of HDAC5 did not significantly affect type I IFN (ISRE)-, type II IFN (GAS)-, or NF-κB-dependent Luc expression in response to cytokine stimulation (IFN-α, IFN-γ, tumor necrosis factor alpha [TNF-α], or interleukin-1β [IL-1β]) (Figures 1A–1D). However, the ISG56 promoter, activated by SeV infection, was significantly diminished in both *HDAC5*$^{-/-}$ cell lines, indicating that HDAC5 is a positive regulator of the IRF3 pathway (Figure 1E). To further explore the IRF3 pathway, the transcription of endogenous IRF3-responsive genes was measured by quantitative reverse-transcription PCR (RT-qPCR) after stimulation with SeV. Consistent with the reporter gene assay, the transcription of two IRF3-responsive genes, *IFN-β* and *ISG56*, was significantly diminished in the absence of HDAC5 (Figures 1F and 1G). To confirm the reduced IRF3 activation in the absence of HDAC5, phospho-IRF3 (p-IRF3) levels were also analyzed after SeV infection. Consistent with the reporter gene assays and RT-qPCR analysis, both HeLa *HDAC5*$^{-/-}$ cells showed diminished p-IRF3 levels compared with parental cells (Figures S1A and S1B). Similar reporter gene assays were performed in parental HEK293T and derivative *HDAC5*$^{-/-}$ cells (referred to as H5KO3 and H5KO4),[12] and these cells responded normally to IFN-α and TNF-α stimulation (Figures 1H and 1I) but were defective in IRF3 activation after SeV infection (Figure 1J). The activation of the IRF3 pathway in these HEK293T cell lines, was also analyzed by measuring p-IRF3 levels after SeV infection. Consistent with the reporter gene assays, *HDAC5*$^{-/-}$ cells showed diminished p-IRF3 levels (Figures 1K and 1L). In summary, four *HDAC5*$^{-/-}$ cell lines from two independent cell types showed a defect in activation of the IRF3 pathway.

Next, the consequence of HDAC5 overexpression was tested. Transient transfection of a plasmid expressing FLAG-tagged HDAC5 enhanced IRF3 activation in HeLa cells (Figure 2A) and HEK293T cells (Figure 2F) in a dose-dependent manner after the cells were stimulated by SeV. To confirm that the defect in IRF3 activation in *HDAC5*$^{-/-}$ cells was due to loss of HDAC5 rather than an off-target effect of the CRISPR-Cas9 system, a complementation assay was performed. Transient transfection of plasmids expressing FLAG-tagged HDAC5 (Figures 2B and 2C), but not HDAC4 (Figures 2D and 2E) or HDAC1 (Figures S1C and S1D), rescued SeV-induced ISG56 promoter-activation in both HeLa *HDAC5*$^{-/-}$ cell lines in a dose-dependent manner, confirming that HDAC5 positively regulates IRF3 activation. Similarly, in both HEK293T *HDAC5*$^{-/-}$ cell lines, expression of HDAC5 (Figures 2G and 2H), but not HDAC4 (Figures 2I and 2J) or HDAC1 (Figures S1E and S1F), rescued ISG56.1 promoter activation after SeV infection. Indeed, HDAC1 overexpression was inhibitory (Figures S1E and S1F).

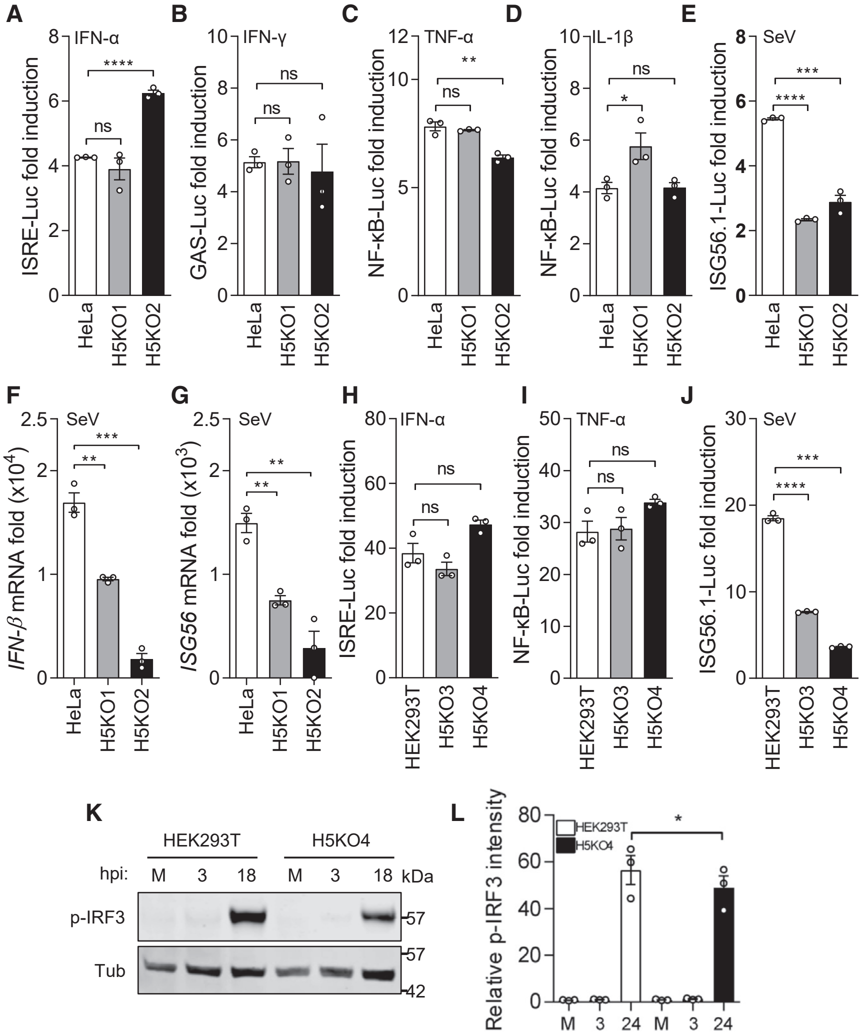

Figure 1. *HDAC5*$^{-/-}$ cells have poor IRF3 activation after SeV infection

(A–E) HeLa *HDAC5*$^{-/-}$ cells have diminished IRF3 response to SeV infection. Parental HeLa and derivative *HDAC5*$^{-/-}$ clones H5KO1 and K5KO2 were seeded in 96-well plates and transfected with 100 ng per well of ISRE-, GAS-, NF-κB-, or ISG56.1-Luc reporter plasmid and 10 ng per well of TK-*Renilla* plasmid overnight. Transfected cells were then stimulated with 1,000 units/mL IFN-α (ISRE-Luc) (A), 250 ng/mL IFN-γ (GAS-Luc) (B), 10 ng/mL TNF-α (NF-κB-Luc) (C), and 100 ng/mL IL-1β (D) (NF-κB-Luc) for 6 h or infected with SeV (ISG56.1-Luc) overnight (E). Cells were then lysed and processed to measure firefly and *Renilla* Luc activity. Firefly Luc was measured and normalized to the *Renilla* Luc control. The fold induction was calculated relative to unstimulated controls. The assays were performed in triplicate, and each experiment was conducted at least three times.

(F and G) RT-qPCR analysis of IRF3-responsive genes in *HDAC5*$^{-/-}$ cells. The same cell lines as in (A) was infected with SeV for 4 h, and mRNA levels of *IFN-β* and *ISG56* were measured by RT-qPCR.

(H–J) HEK293T-derived *HDAC5*$^{-/-}$ cells respond poorly to SeV infection. As in (A), parental HEK293T and *HDAC5*$^{-/-}$ clones H5KO3 and H5KO4 were transfected with ISRE, NF-κB, or ISG56.1-Luc reporter plasmid and TK-*Renilla* plasmid overnight. Cells were then stimulated with 1,000 units/mL IFN-α (H) and 20 ng/mL TNF-α (I) or infected with SeV (J), and the fold induction of the Luc was calculated as in (A)–(E).

(K and L) Immunoblot showing SeV-induced *p*-IRF3 levels. The same cells as in (C) were infected with SeV, collected at different times p.i., and analyzed by immunoblotting (K) for levels of phosphorylated IRF3 (p-IRF3) and α-tubulin. (L). The p-IRF3 level was calculated relative to α-tubulin (Tub) ± SEM from 3 independent experiments.

ns, not significant. *$p < 0.05$, **$p < 0.01$, ***$p < 0.001$, ****$p < 0.0001$.

Mapping where HDAC5 activates the IRF3 pathway

Activation of IRF3 may be induced by factors that act at different stages in the pathway, such as retinoic acid-inducible gene-I (RIG-I), mitochondrial antiviral signaling protein (MAVS), TRIF, and p-IRF3. To investigate the stage(s) at which HDAC5 promotes IRF3 activation, HEK293T cells and derivative *HDAC5*$^{-/-}$ cells (H5KO3 and H5KO4) were transiently transfected with plasmids expressing the FLAG-tagged RIG-I caspase activation and recruitment domain (CARD) (RIG-I-CARD), MAVS, or TRIF lacking the receptor interacting protein (RIP) homotypic interaction domain (TRIFΔRIP) together with ISG56.1-Luc and TK-*Renilla*. In the HEK293T *HDAC5*$^{-/-}$ cell lines, IRF3 activation induced by TRIFΔRIP was reduced compared with parental HEK293T cells, whereas activation by RIG-I-CARD and MAVS was unchanged in *HDAC5*$^{-/-}$ cell lines (Figures 3A–3C). Immunoblotting showed consistent expression levels of MAVS and TRIFΔRIP in the different cell lines (Figures 3D and 3E), but the level of RIG-I-CARD was not shown due to its low expression. Further analysis also showed that overexpression of HDAC5 in HEK293T cells enhanced TRIFΔRIP- but not IRF3-5D-mediated IRF3 pathway activation (Figures S2A and S2B). Immunoblotting showed the expression of the indicated proteins (Figure S2C). To confirm the role of HDAC5 in regulating the IRF3 pathway, a similar complementation assay as in Figure 2B was performed, except that the ISG56.1 promoter was activated by overexpression of TRIFΔRIP instead of SeV infection. Expression of FLAG-tagged HDAC5 in both HEK293T and H5KO3 cells upregulated TRIF-mediated IRF3 pathway activation (Figures 3F and 3G). In contrast, HDAC1 (Figure S2D) and HDAC4 (Figure 3H) downregulated the TRIF-mediated ISG56.1 reporter expression. This is consistent with previous studies demonstrating that overexpression of HDAC4 negatively regulates the IRF3 pathway.[11,17] Collectively, these observations indicate that HDAC5 positively regulates IRF3 activation and does so at the level of TRIF-mediated activation.

Histidine 885 of mouse HDAC5 is essential for the HDAC enzymatic activity,[29] and given the high conservation of the active site of HDAC5 among different mammalian species

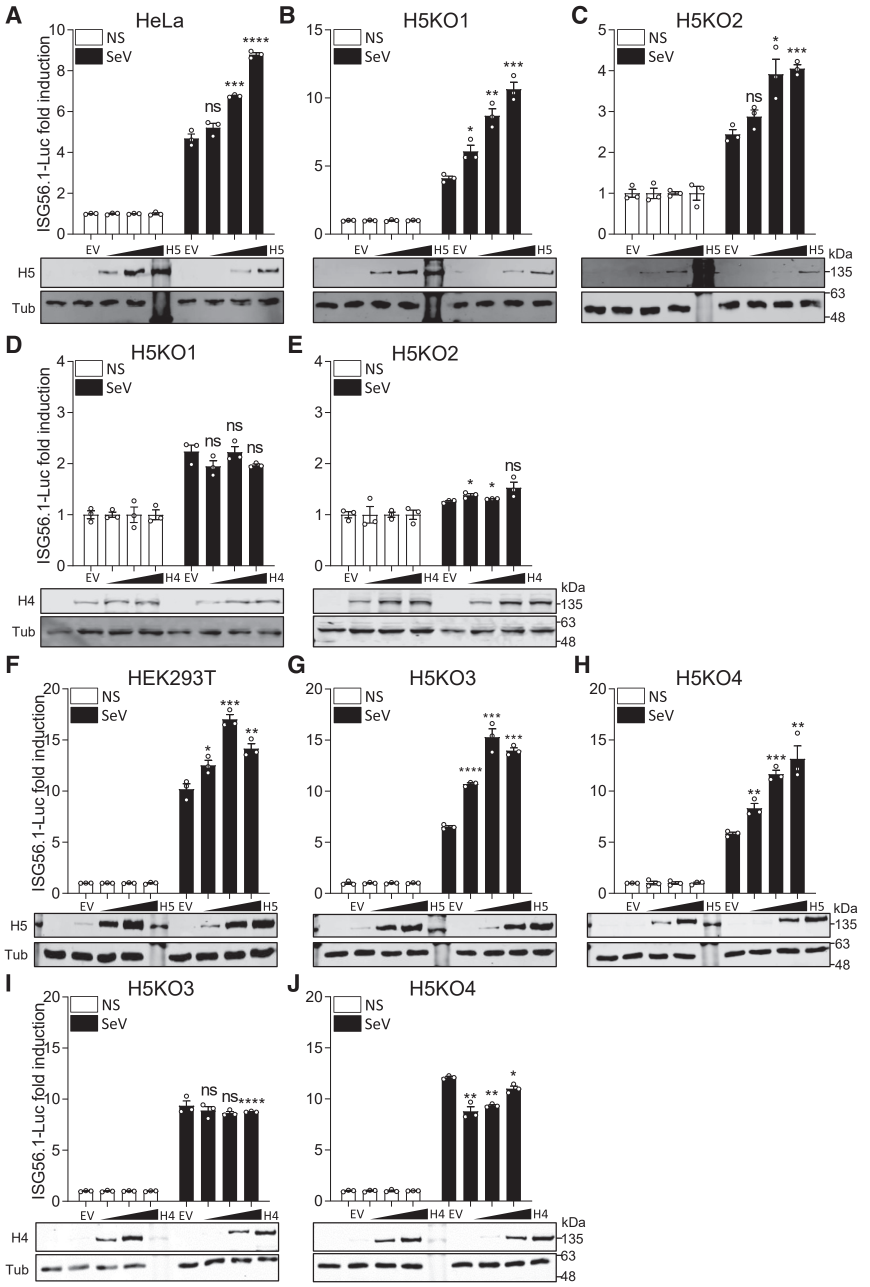

Figure 2. HDAC5 rescues IRF3 activation in *HDAC5*$^{-/-}$ cells

(A–C) Expression of HDAC5 enhances IRF3 activation in HeLa and derivative *HDAC5*$^{-/-}$ cells. *HDAC5*$^{-/-}$ clones H5KO1 or H5KO2 or parental HeLa cells were co-transfected with ISG56.1-Luc and TK-*Renilla* reporter plasmids, along with a FLAG-tagged HDAC5-expressing vector at 0, 10, 50, or 100 ng per well, and then infected with SeV overnight. Cell lysates were collected and analyzed as in Figure 1.

(D and E). Expression of HDAC4 does not rescue IRF3 activation in the *HDAC5*$^{-/-}$ cells. The same reporter assays as in (B) and (C) were performed with overexpression of FLAG-tagged HDAC4 (H4) or HDAC1 (H1) (Figures S1A and S1B).

(F–J). Expression of HDAC5, but not HDAC1 or HDAC4, enhances IRF3 activation in HEK293T and derivative *HDAC5*$^{-/-}$ cells. The same reporter assays as in (A) were performed in parental HEK293T or derivative *HDAC5*$^{-/-}$ clones H5KO3 and H5KO4 with overexpression of FLAG-tagged HDAC5 (F–H), HDAC4 (I and J), or HDAC1 (Figures S1C–S1F).

Data are presented as mean ± SEM, n ≥ 3 independent experiments. *$p < 0.05$, **$p < 0.01$, ***$p < 0.001$, ****$p < 0.0001$. The expression of HDAC5-FLAG (H5) or HDAC4-FLAG and Tub were measured by immunoblotting and are shown underneath the reporter gene assay. The positions of molecular mass markers in kiloDaltons are shown on the right.

(Figure 3I), the same amino acid (aa) substitution of human HDAC5 (H5-H893F) was introduced by site-directed mutagenesis. This mutant complemented TRIF-activated ISG56.1 reporter expression in a dose-dependent manner (Figure 3J) just like wild-type (WT) HDAC5, indicating that the enzymatic activity of HDAC5 is not required for upregulating IRF3 activation.

HDAC5 restricts orthopoxvirus replication and is targeted for proteasome degradation

HDAC5 is targeted by VACV strain Western Reserve (WR) for proteasomal degradation.[12] To determine whether other strains of VACV also degrade HDAC5, human fetal foreskin fibroblasts (HFFFs) were infected with VACV strains WR, Lister, or Copenhagen (Cop), and the level of HDAC5 was analyzed by immunoblotting at different times post infection (pi). This showed that HDAC5 was also downregulated by VACV strains Lister and Cop, with Lister being the most effective (Figure S3A). To investigate whether other orthopoxviruses also down-regulate HDAC5, HFFFs or HEK293T cells were infected with CPXV strain Brighton Red (CPXV-BR), RPXV, CMLV, MPXV, or elephantpox virus (a CPXV strain; CPXV-E), and cell extracts were analyzed by immunoblotting. As for VACV strains, all of these orthopoxviruses downregulated HDAC5, and this downregulation was rescued by the proteasome inhibitor MG132, indicating that HDAC5 is targeted for proteasomal degradation (Figures 4A–4D; Figure S3B). Further analysis showed that MPXV also degraded HDAC5 in HeLa cells (Figure S3C).

HDAC5 acts as a restriction factor for VACV and HSV-1,[12] and so its activity was also tested against other orthopoxviruses. To do this, parental HeLa and derivative *HDAC5*$^{-/-}$ cells were infected with CPXV-BR, RPXV, CMLV, and CPXV-E, and the yields of infectious virus were determined. *HDAC5*$^{-/-}$ cells yielded more infectious virus, showing that HDAC5 restricts replication

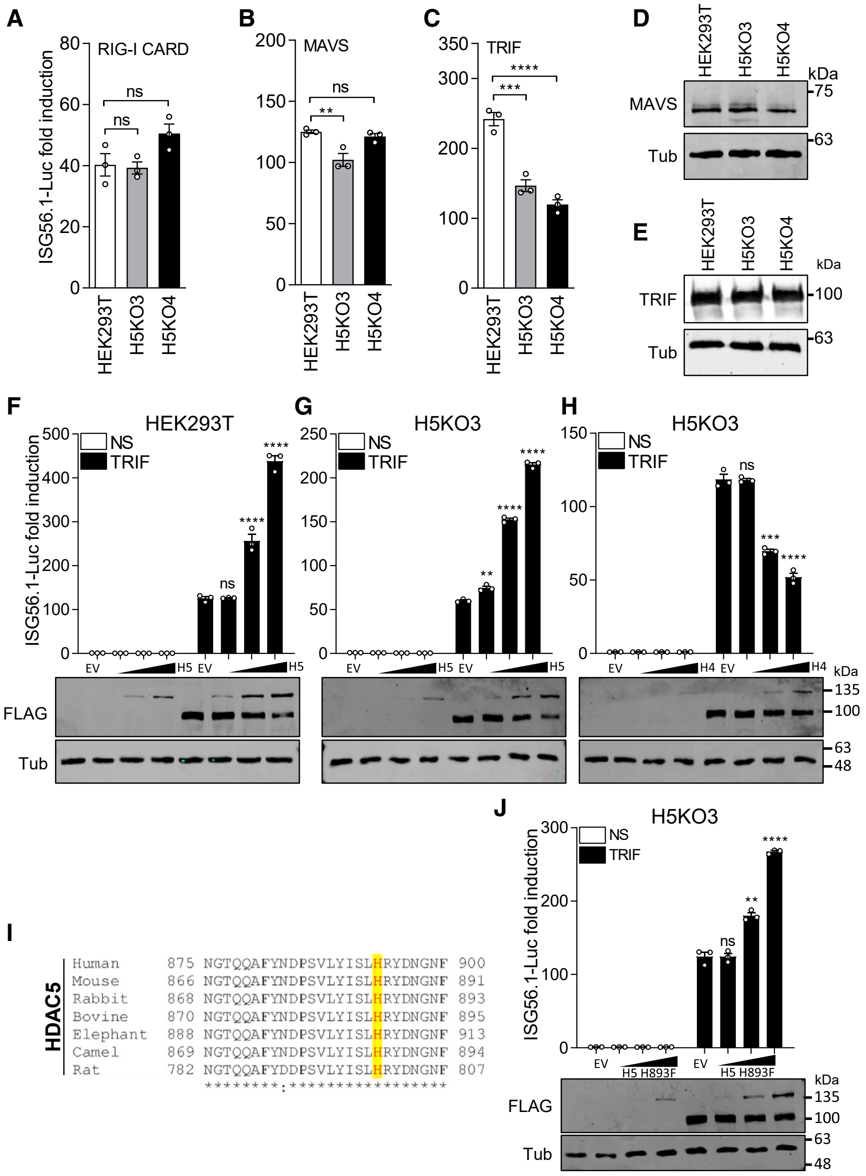

Figure 3. HDAC5 enhances TRIF-mediated IRF3 activation

(A–C) Activation of the IRF3 pathway by TRIF, but not RIG-I or MAVS, is diminished in *HDAC5*$^{-/-}$ cells. Parental HEK293T and *HDAC5*$^{-/-}$ cells were co-transfected with ISG56.1-Luc, TK-*Renilla* Luc reporter plasmids, and plasmids expressing either RIG-I CARD, MAVS, or TRIFΔRIP to activate the IRF3 pathway. Cell lysates were collected 18 h after transfection, and the ISG56.1-Luc reporter expression was analyzed as in Figure 1.

(D and E). Immunoblots showing FLAG-tagged MAVS, TAP-tagged TRIFΔRIP, and endogenous Tub expression levels in cell lysates from (B) and (C), respectively.

(F–H). Expression of HDAC5, but not HDAC4, enhances TRIF-mediated IRF3 activation in parental HEK293T cells and *HDAC5*$^{-/-}$ cells. Parental HEK293T (F) or H5KO3 (G and H) cells were transfected overnight with plasmids expressing ISG56.1-Luc, TK-*Renilla*, TAP-TRIFΔRIP, and HDAC5-FLAG (F and G) or HDAC4-FLAG (H). The HDAC5-FLAG- or HDAC4-FLAG-expressing plasmids were transfected at 0, 10, 50, or 100 ng per well. The relative ISG56.1-Luc reporter expression was analyzed as in Figure 1.

(I). Alignment of HDAC5 sequences from human, mouse, rabbit, bovine, elephant, camel, and rat. A conserved histidine that is essential for HDAC activity is highlighted in yellow.

(J) HDAC5 enzymatic activity is not needed to enhance TRIF-mediated IRF3 activation; as for (G), except that HDAC5-FLAG was replaced with HDAC5-H893F-FLAG (H5 H893F), a plasmid expressing an enzymatically inactive mutant.

In (F)–(H) and (J) immunoblots for FLAG-tagged proteins and Tub are shown beneath the reporter gene assays. Data are presented as mean ± SEM, n ≥ 3 independent experiments. **p < 0.01, ***p < 0.001, ****p < 0.0001.

of these orthopoxviruses (Figures 4E–4H). To investigate this further, enhanced yellow fluorescent protein (EYFP) or HDAC5 was overexpressed inducibly in U2OS cells (Figure S3D) and then infected with the indicated orthopoxviruses. Overexpression of HDAC5, but not EYFP, reduced plaque size for CPXV-BR (Figures 4I and 4J) and reduced the yield of infectious virus for CPXV-BR, CMLV, CPXV-E, and RPXV 2 days after infection (Figures 4K–4M and S3E).

Orthopoxvirus protein C6 interacts with HDAC5

Previously, VACV WR protein C6 has been shown to be necessary and sufficient to induce the proteasomal degradation of HDAC5.[12] To determine whether these proteins co-precipitate during infection, cells were infected with VACV WR expressing tandem affinity purification (TAP)-tagged C6 (vTAP-C6) or N1 (vTAP-N1), another VACV Bcl-2-like protein with functions in regulation of innate immune signaling.[30,31] The TAP tag contains 2 copies of the Strep tag and 1 copy of the FLAG epitope.[32] At 3 or 6 hpi, TAP-tagged proteins were affinity purified from cell lysates using Strep-Tactin beads. Immunoblotting showed that endogenous HDAC5 co-precipitated with TAP-C6, but not TAP-N1, during infection (Figure 5A). No other virus protein was needed because TAP-C6 expressed ectopically co-precipitated with HDAC5, whereas protein VACV TAP-N1 did not (Figure 5B).

Given that HDAC5 is degraded by RPXV, CPXV-BR, CPXV-E, CMLV, and MPXV_CVR_S1, we investigated whether the C6 orthologs from these poxviruses also co-precipitate with HDAC5. T-REx-293 cell lines were prepared to inducibly express TAP-tagged C6 orthologs from RPXV, CPXV-BR, CPXV-E, CMLV, MPXV clade I (Zaire) and clade II (Glasgow_2022), and VARV as well as TAP-tagged N1 from VACV WR. Lysates from induced cells were subjected to affinity purification, and immunoblotting revealed that endogenous HDAC5 co-precipitated

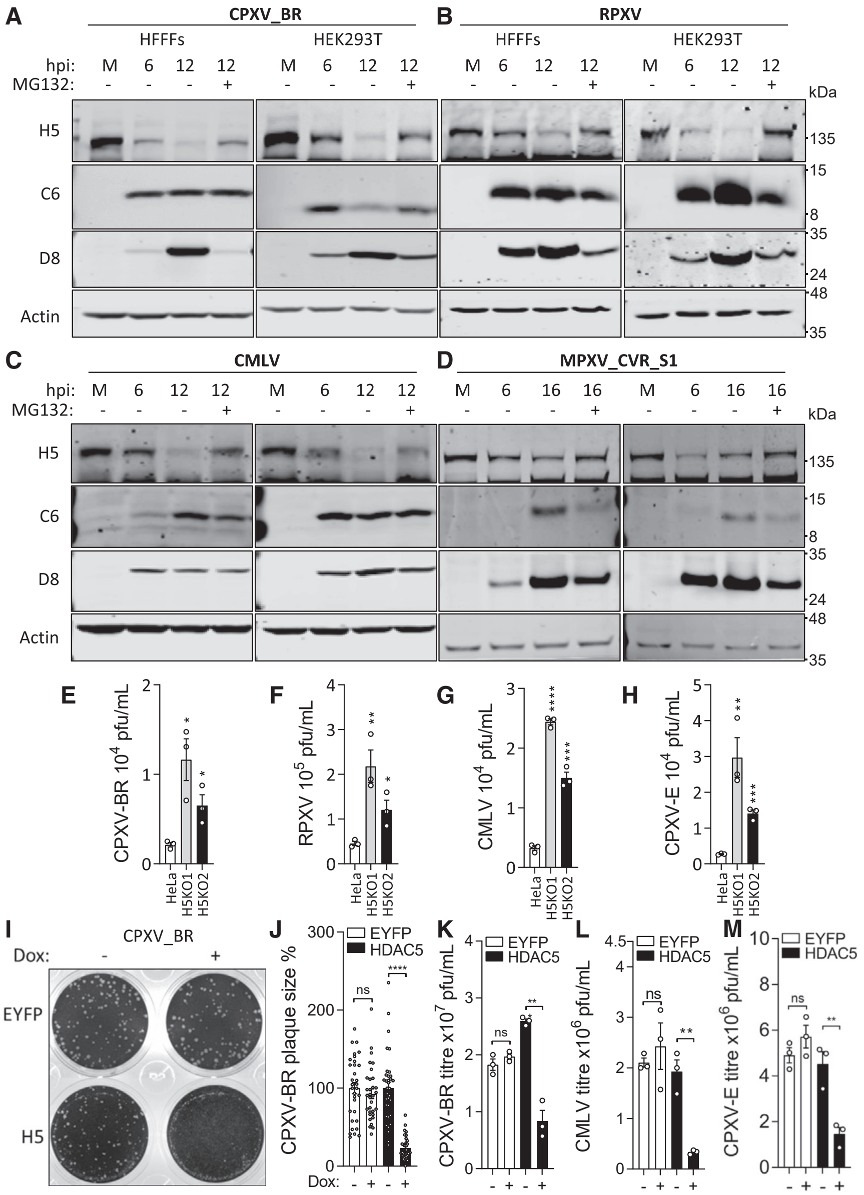

Figure 4. HDAC5 restricts the replication of multiple orthopoxviruses and is targeted for degradation by these viruses

(A–D) CPXV-BR, RPXV, CMLV, and an MPXV strain isolated from 2022 (MPXV_CVR_S1) degrade HDAC5 during infection. HFFFs or HEK293T cells were infected with CPXV-BR (A), RPXV (B), CMLV (C), or MPXV_CVR_S1 (D) at 5 plaque-forming units (PFUs) per cell for 2 h before the inoculum was removed and replaced with fresh medium or medium supplemented with 20 μM MG132. The infected cells were collected at different times p.i. as indicated, and proteins were analyzed by immunoblotting using the indicated antibodies. The positions of molecular mass markers in kiloDaltons are indicated on the right.

(E–H) Replication of orthopoxviruses is enhanced in *HDAC5*$^{-/-}$ cells. Parental HeLa and derivative *HDAC5*$^{-/-}$ cells were infected with the indicated orthopoxviruses at MOI = 0.01. Two days p.i., the supernatant and infected cells were collected, and infectious virus was titrated on BSC-1 cells. n = 3 independent experiments.

(I and J) Expression of HDAC5 in U2OS cells restricts CPXV-BR plaque size. U2OS.TetR.EYFP or U2OS.TetR.HDAC5-FLAG cells were mock-induced or induced with 100 ng/mL doxycycline (dox) for 18 h and then infected with 100 PFUs of CPXV-BR per well. After 2 h of infection, the inoculum was removed and replaced with 1.0% carboxymethyl cellulose (CMC) in DMEM (+ dox where indicated). Plaque images were recorded after the infected cells were fixed with 4% paraformaldehyde and stained with toluidine blue. Representative images are shown in (I). Plaque sizes in the presence (+) or absence (–) of dox were quantified and compared (J). n ≥ 32/condition.

(K–M) Expression of HDAC5 in U2OS cells restricts CPXV-BR, CMLV, and CPXV-E replication. U2OS.TetR.EYFP or U2OS.TetR.HDAC5-FLAG cells were mock-induced or induced with dox for 18 h and then infected with CPXV-BR, CMLV, and CPXV-E at 0.01 PFUs per cell for 2 days (CPXV-BR) or 3 days (CMLV and CPXV-E). The culture medium and infected cells were collected, and virus titer was quantified by plaque assay on BSC-1 cells.

Data are presented as mean ± SEM, n ≥ 3 independent experiments. $^{**}p < 0.01$, $^{***}p < 0.001$, $^{****}p < 0.0001$.

with all listed orthopoxvirus C6 proteins but not N1 (Figure 5C). Next, the level of endogenous HDAC5 was quantified in the cell lines that had or had not been induced to express the C6 protein and was normalized to actin (Figures 5D and S4A). This showed that all the C6 proteins, but not N1, induced degradation of HDAC5 outwith virus infection. These observations suggests that MPXV_Zaire and VARV are also likely to target HDAC5 for degradation via C6 proteins during infection. Considering that C6 is responsible for degrading HDAC5 to evade HDAC5-mediated restriction, the absence of C6 might be expected to amplify the differences in viral yield between control and *HDAC5*$^{-/-}$ cells. To examine this possibility, parental HeLa, H5KO1, and H5KO2 cells were infected with vΔC6 or WT VACV, and the virus yield was measured. The lack of C6 slightly but significantly magnified the differences in viral yield compared with the WT VACV (Figure S4B).

Class II HDAC proteins contain an N-terminal domain that regulates calcium signals and mediates interactions with transcription factors and cofactors, while the C-terminal domain has HDAC enzymatic activity.[33] To determine which domain of HDAC5 interacts with C6, FLAG-tagged full-length HDAC5, an N-terminal truncation (aa 1–684, HDAC5N), or a C-terminal truncation mutant (aa 684–1122, HDAC5C) were co-expressed in human cells with TAP-C6. Affinity-purified TAP-C6 was found to co-precipitate with HDAC5 and HDAC5N but not HDAC5C (Figure S4C), indicating that the interaction occurs via the HDAC5 N-terminal domain. To investigate whether C6 interacts directly with HDAC5, recombinant proteins were expressed *in vitro* using

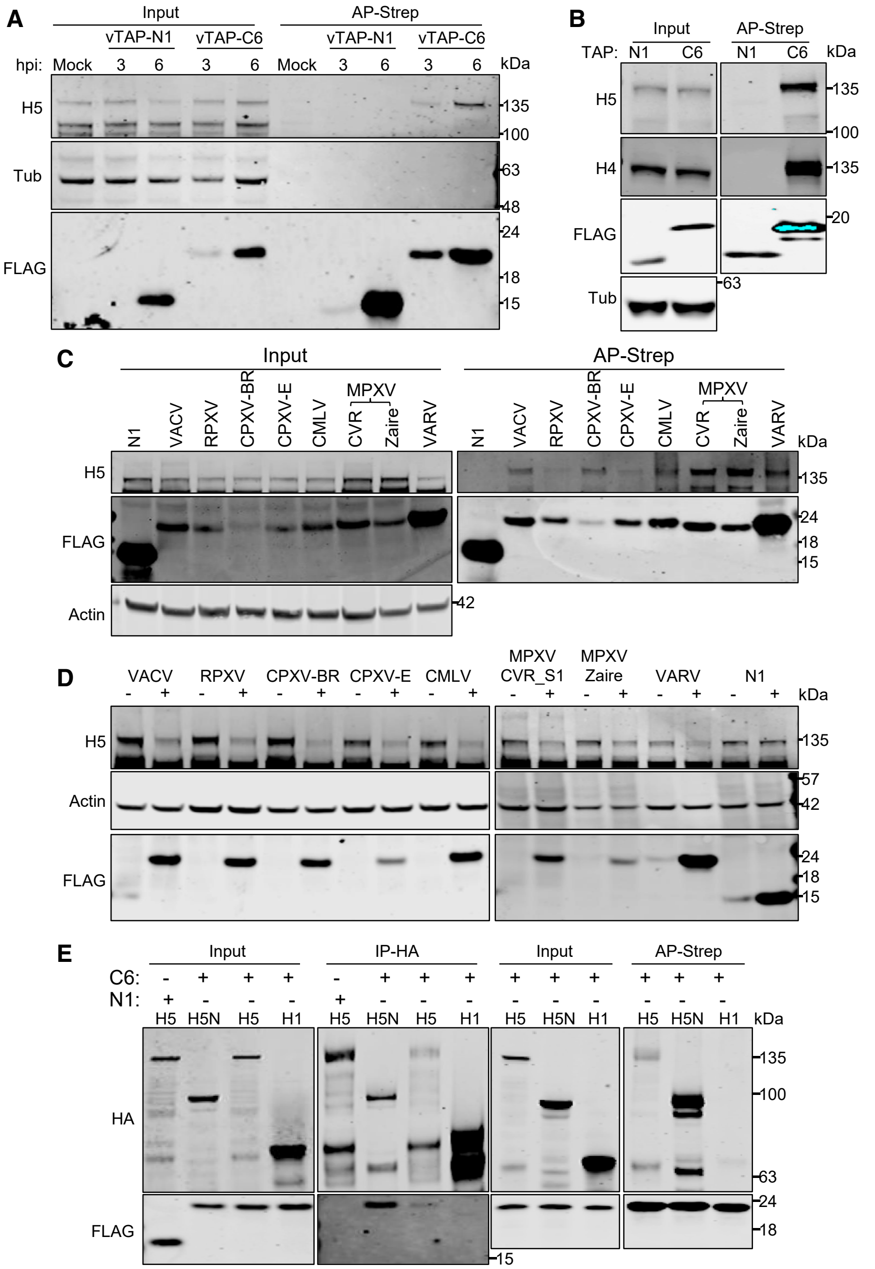

Figure 5. The C6 protein from orthopoxviruses interacts with and induces degradation of HDAC5

(A) VACV C6 co-precipitates with HDAC5 during infection. HEK293T cells were mock infected or infected with VACV expressing either a TAP-tagged N1 (vTAP-N1) or C6 (vTAP-C6) at 5 PFU per cell. At 3 h or 6 h p.i., the cells were washed with PBS twice, lysed, and clarified by centrifugation, and TAP-tagged proteins were subjected to affinity purification (AP) by Strep-Tactin. Input (left), or affinity-purified proteins were analyzed by immunoblots with antibodies as indicated.

(B) VACV C6 co-precipitates with HDAC5 outwith infection. HEK293T cells were transfected with plasmids expressing TAP-tagged N1 or C6 overnight. The cells were then lysed, and samples were analyzed as in (A).

(C) Orthopoxvirus protein C6 co-precipitates with HDAC5. T-REx-293 cells inducibly expressing TAP-tagged VACV N1; C6 from VACV, RPXV, CPXV-BR, CPXV-E, CMLV, MPXV_Zaire, MPXV_CVR_S1; or codon-optimized C6 of VARV were induced with 100 ng/mL dox for 24 h. The cell lysates were collected, and TAP-tagged proteins were affinity purified via the streptavidin epitope. Input (left) or affinity-purified proteins (right) were analyzed as in (A).

(D) Orthopoxvirus protein C6 induces HDAC5 degradation outwith infection. The cells in (C) were mock induced or induced with dox for 24 h before cell lysates were prepared and analyzed by immunoblotting.

(E) C6 interacts directly with HDAC5 via the N-terminal domain. TAP-tagged N1 or C6 was co-expressed with HA-tagged HDAC1, HDAC5, or H5N using a wheat germ cell-free transcription and translation system. Left and center left: HA-tagged proteins were immunoprecipitated, and the purified proteins were analyzed by immunoblotting using antibodies as indicated. Right and center right: the same co-expression samples were prepared without N1, TAP-C6 was precipitated, and purified proteins were analyzed as described above.

In all immunoblots, the positions of molecular mass markers are shown in kiloDaltons on the right.

a wheat germ transcription and translation system. Hemagglutinin (HA)-tagged HDAC5, HDAC5N, or HDAC1 was co-expressed with TAP-tagged N1 or C6 and immunoprecipitated via anti-HA-conjugated beads. TAP-C6 co-precipitated with HA-HDAC5N (strongly) and HA-HDAC5 (weakly) but not HA-HDAC1, showing that C6 does not require an additional mammalian protein to co-precipitate with HDAC5 and so likely interacts directly with the HDAC5 N-terminal domain (Figure 5E, left and center left). A reciprocal co-immunoprecipitation (coIP) confirmed that FLAG-tagged C6 interacted directly with HA-HDAC5 and HDAC5N but not HDAC1 (Figure 5E, right and center right). Given the structural similarity between HDAC4 and HDAC5, it is conceivable that C6 interacts directly with both proteins via their N-terminal region. To explore this, glutathione S-transferase (GST)-tagged VACV C6 was expressed and purified from *E. coli*. Simultaneously, myc-tagged HDAC4, H4N, HDAC5, H5N, and HSV-1 protein Vsp18 were expressed *in vitro* using the wheat germ expression system. Consistent with mammalian cell lines and the wheat germ expression system, GST-C6 co-precipitated with HDAC4 and HDAC5 via their N-terminal region, while no interaction was observed with Vps18 (Figure S4D). The use of purified recombinant protein also confirmed that C6 and HDAC5 interact directly. To map the region of HDAC5 mediating the interaction in more detail, co-precipitation of the purified

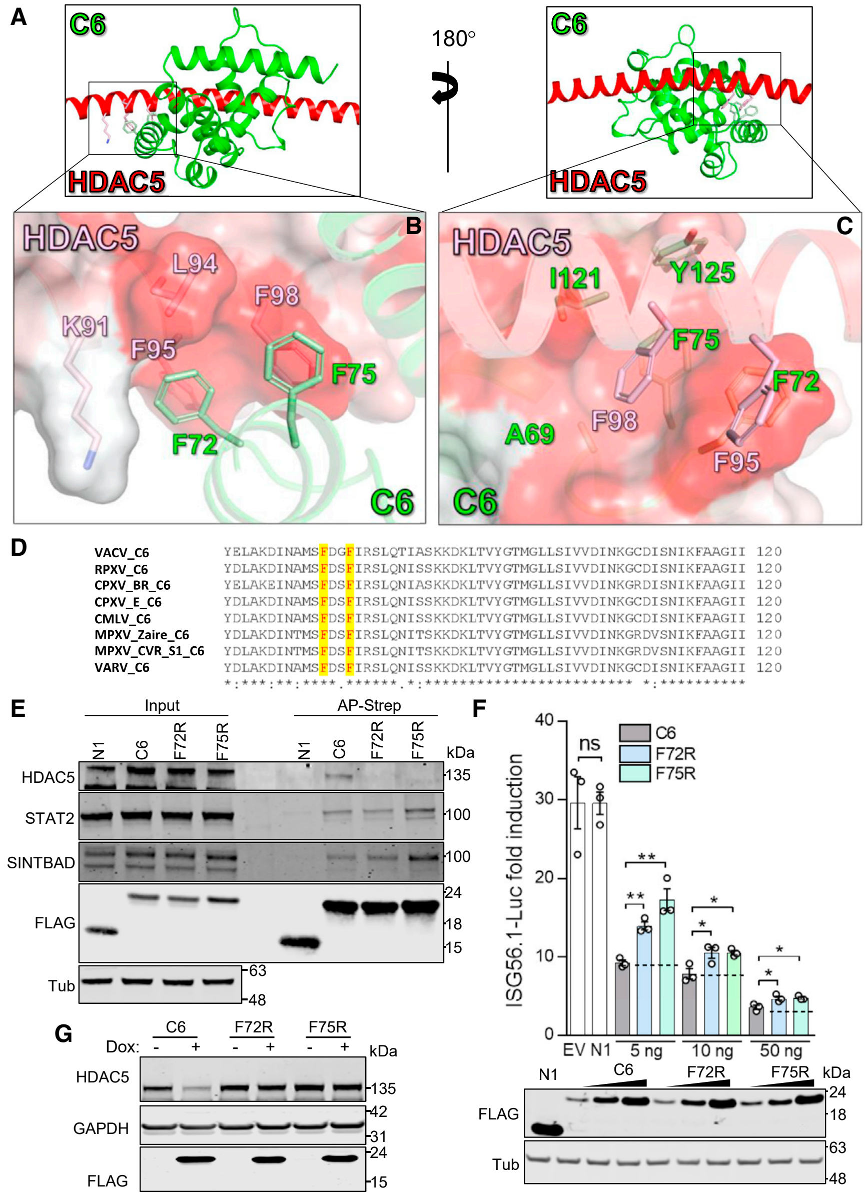

Figure 6. C6 F72 and F75 are needed for interaction with HDAC5

(A–C) AFM prediction of key interactions at the binding interface between C6 and HDAC5.

(A) Ribbon diagrams showing the AFM-predicted complex structures of HDAC5 (red) and C6 (green).

(B) F72 and F75 of C6 bind into a hydrophobic cleft formed by K91, L94, F95, and F98 of HDAC5. C6 is shown as a ribbon in green. HDAC5 is shown both as a ribbon diagram (red) and surface, which is colored by residue hydrophobicity from red (hydrophobic) to white (polar).

(C) Residue F98 of HDAC5 (pale pink) binds into a hydrophobic cleft formed by A69, F72, F75, I121, and Y125 of C6 (green), while F98 of HDAC5 is pointing away from the cleft. HDAC5 is shown as a ribbon diagram in red. C6 is shown both as a ribbon diagrams (green) and surface, which is colored by residue hydrophobicity from red (hydrophobic) to white (polar). Side chains of the residues are shown as sticks and are colored pale pink (HDAC5) and green (C6), respectively, while nitrogen is colored blue.

(D) Aa sequence alignment of the orthopoxvirus C6 proteins tested in Figure 5. The positions of VACV F72 and F75 and the conserved aas in other poxviruses are highlighted in yellow.

(E) C6 F72 and F75 are needed for interaction with HDAC5. TAP-tagged N1, C6, or C6 mutant F72R or F75R was expressed in HEK293T cells by transfection. TAP-tagged proteins were affinity purified by Strep-Tactin and analyzed by immunoblotting with the indicated antibodies.

(F) C6 mutants F72R and F75R restrict IRF3 activation less efficiently than WT C6. An ISG56.1-Luc reporter gene assay was performed with the indicated levels of TAP-tagged C6, C6 F72R, or F75R. Empty vector (EV) and TAP-tagged N1 were included as negative controls. The cells were stimulated with SeV for 16 h and then collected for measurement of firefly and *Renilla* Luc activity. Immunoblots below the graph show expression of TAP-tagged proteins and Tub.

(G) C6 mutants F72R and F75R do not induce HDAC5 degradation. T-REx-293 cells inducibly expressing TAP-tagged VACV C6 or C6 mutants F72R or F75R were mock induced or induced with dox for 24 h before cell lysates were prepared and analyzed by immunoblotting.

Data are presented as mean ± SEM, n ≥ 3 independent experiments. *p < 0.05, **p < 0.01. In (E)–(G), the positions of molecular mass markers are shown in kiloDaltons on the right.

GST-C6 and myc-tagged truncation mutants of HDAC5N, expressed in the wheat germ expression system, was tested. HDAC5N mutants lacking aa 1–52 and 1–67 interacted with C6, but a mutant lacking 1–170 failed to do so (Figure S4E). Notably, this region of HDAC5 is needed for the formation of homodimers and higher-order oligomers.[24]

Given that the structure of HDAC5N is known,[24] VACV protein C6 is predicted to have a Bcl-2 fold,[34] and HDAC5 and C6 interact directly, models of C6-HDAC5 complexes were generated using the greatly improved protein structure prediction program[35] AlphaFold Multimer (AFM).[36] Predictions were performed using VACV WR C6 in complex with HDAC5 aa 67–170, aa 1–650, or full-length HDAC5 using default parameters. All three AFM searches predicted that C6 interacts with aa 92–118 of HDAC5 and that the interaction site represents a hydrophobic pocket formed by three phenylalanine residues: HDAC5 F98 and C6 F72 and F75 (Figures 6A–6C). F72 and F75 are highly conserved among C6 proteins from different orthopoxviruses (Figure 6D), and so, to test their importance for interaction with HDAC5, these residues were mutated individually to arginine (F72R and F75R) in the FLAG-tagged VACV WR C6 protein. Expression of these mutants in mammalian cells showed that

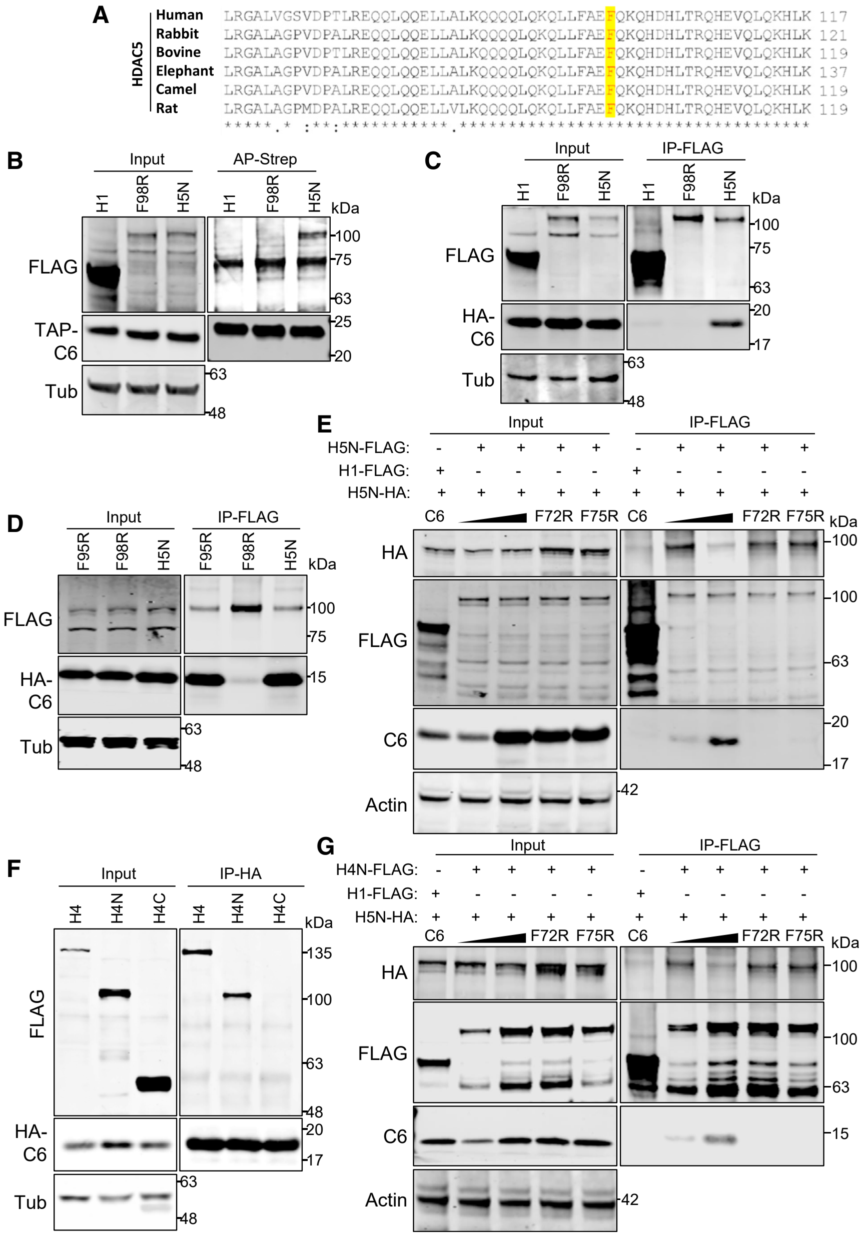

Figure 7. HDAC5 F98 is needed for interaction with C6

(A) Aa sequence alignment of HDAC5 proteins from different species. The position of human HDAC5 F98 and the corresponding conserved aas in other species are highlighted in yellow.

(B and C) HDAC5 F98 is needed to interact with C6. TAP-tagged C6 and FLAG-tagged HDAC1, HDAC5-N, or HDAC5-N with F98R mutation were co-expressed in HEK293T cells by transfection and affinity purified via Strep-Tactin. Input (left) and purified proteins (right) were analyzed by immunoblotting with the indicated antibodies.

(C) Reciprocal IP assay of (B). The same cellular proteins as in (B) were co-expressed with HA-tagged C6 in HEK239T cells by transfection. FLAG-tagged HDAC1, HDAC5N, or HDAC5N-F98R was purified via the FLAG epitope. Input (left) and purified proteins (right) were analyzed as described in (B).

(D) HDAC5 F95 is not needed for C6/HDAC5 interaction; as in (C), except that HDAC1 was replaced with HDAC5-N mutant F95R. FLAG-tagged proteins were purified, and the protein samples were analyzed by immunoblotting using antibodies as indicated.

(D) C6 interrupts HDAC5 homodimerization. HA- and FLAG-tagged HDAC5-N were co-expressed in HEK293T cells together with increasing amounts of HA-tagged C6 or C6 mutant F72R or F75R. HDAC1 was included as a negative control. FLAG-tagged proteins were purified and analyzed by immunoblotting with the indicated antibodies.

(E) HDAC4 interacts with C6 via the N-terminal domain. HA-tagged C6 was co-expressed with FLAG-tagged full-length HDAC4, N-terminal (H4N, 1–650), or C-terminal (H4C, 651–1084) of HDAC4 in HEK293T cells by transfection and purified via the HA tag. Purified proteins were analyzed as described in (A).

(F) C6 outcompetes HDAC5 for heterodimerization with HDAC4; as in (D), except that FLAG-tagged H4N was purified and analyzed by immunoblotting with the indicated antibodies.

In all immunoblots, the positions of molecular mass markers are shown in kiloDaltons on the right.

the proteins were stable and folded normally because they were still able to co-precipitate with the known C6 partners similar to NAP1 TBK1 adaptor (SINTBAD)[37] and STAT2[38] (Figure 6E). In contrast, and consistent with the AFM prediction, these mutants did not co-precipitate with endogenous HDAC5 (Figure 6E). Three other assays were used to assess the functionality of the C6 F72R and F75R mutants. The first was the subcellular distribution of FLAG-tagged HDAC5. In U2OS cells, immunofluorescence (IF) showed that, when expressed individually, FLAG-HDAC5 was nuclear, while C6 was cytoplasmic (Figures S5A and S5B), but when expressed together, HDAC5 was translocated to the cytoplasm (Figures S5A, top row, and S5B, top two rows). In contrast, in cells expressing either C6 F72R or F75R, HDAC5 remained in the nucleus. As a control, VACV protein N1 did not affect HDAC5 localization (Figure S5A, bottom row). Second, a reporter gene assay measuring activation of the IRF3 pathway revealed that C6 F72R and C6 F75R were less efficient at inhibiting pathway activation than the WT protein despite equivalent expression levels (Figure 6F), showing that targeting HDAC5 by C6 contributes to the inhibition of IRF3 pathway activation. Note that C6 F72R and F75R still partially inhibit the IRF3 pathway, likely due to its ability to co-immunoprecipitate with proteins acting downstream, such as similar to NAP1 TBK1 adaptor (SINTBAD).[37] Third, T-REx-293 cell lines, which inducibly express VACV C6, induce degradation of endogenous HDAC5. However, this degradation was not observed

with C6 F72R or F75R (Figures 6G and S5C). Thus, the above experiments corroborate the AFM-prediction that C6 residues F72 and F75 are important for interaction with HDAC5 and show that this interaction is needed for the re-localization of HDAC5 to the cytoplasm and for the complete inhibition of the IRF3 pathway.

F98 is highly conserved in HDAC5 proteins from different species that can be infected by orthopoxviruses (Figure 7A) and is predicted to contribute to the interaction between HDAC5 and C6. To test this prediction, HDAC5N mutant F98R was prepared and analyzed. TAP-C6 co-precipitated with FLAG-HDAC5N but not with F98R or HDAC1 (Figure 7B), and the failure of HDAC5N F98R to co-immunoprecipitate with C6 was confirmed by reciprocal IP (Figure 7C). As a control, an adjacent conserved phenylalanine (F95) was also mutated (F95R), but this mutant interacted with C6, similar to WT HDAC5N (Figure 7D). These results indicate that AFM accurately identified HDAC5 F98 as a key residue to mediate the interaction with protein C6.

Previous studies have shown that F98 also mediates HDAC5 homodimerization.[24] Therefore, since C6 interacts with HDAC5 via F98, C6 might compete with HDAC5 and prevent homodimerization. To investigate this, FLAG- and HA-tagged HDAC5N were co-expressed with increasing amounts of HA-tagged C6, with TAP-C6-F72R and TAP-C6-F75R used as controls. HDAC1 and HDAC5N were purified via the FLAG epitope, and the purified proteins were analyzed by immunoblotting. This showed that C6, but not C6-F72R or C6-F75R mutants, greatly diminished the homodimerization of HDAC5N (Figure 7E).

Given that the N-terminal domain of class II HDACs is highly conserved (Figure S5D), and C6 interacts directly with HDAC4 via its N terminus (Figure S4D), it was investigated whether C6 interacts with HDAC4 by F93, which is the counterpart of F98 on HDAC5. The results showed that C6 co-precipitated with HDAC4N (Figure 7F), but the interaction was not abolished by mutation at F93 (Figure S5E). This suggests the presence of an additional C6 binding site on HDAC4. Additional mapping of the region of HDAC4 needed for interaction with C6 via the production of N-terminal truncation mutants in the wheat germ expression system (Figure S5F) showed that myc-HDAC4 fusion proteins lacking the dimerization domain still interacted with purified GST-C6 (Figure S5F), consistent with the presence of a second interaction domain within the HDAC4 N-terminal domain. Given that HDAC4 and HDAC5 share high structural similarity in their conserved N-terminal domain and form a heterodimer via this domain, it was hypothesized that C6 may also interfere with the HDAC4/5 heterodimerization by interacting with F98 on HDAC5. To test this hypothesis, a competition assay was undertaken as in Figure 7E, except that FLAG-tagged HDAC4N was used. The results showed that C6 prevents the heterodimerization of HDAC4 and HDAC5, whereas C6 F72R and F75R could not (Figure 7G). These findings confirm that HDAC5 F98 mediates interaction with C6, HDAC5 homodimerization, and heterodimerization with HDAC4 and suggest the presence of a second C6 binding site on HDAC4.

DISCUSSION

This study follows the observations that, during VACV infection of human fibroblasts, VACV protein C6 induces the proteasomal degradation of HDAC5 and that HDAC5 functions as a restriction factor for both VACV and HSV-1 by an unknown mechanism.[12] To address how HDAC5 is antiviral, we report here that HDAC5 promotes stimulation of innate immunity by enhancing activation of the IRF3 pathway. Activation of the IRF3 pathway is crucial for controlling the invasion of pathogens in host cells. Certain pattern recognition receptors (PRRs), including RIG-I/DDX58, MDA5, and cyclic GMP-AMP synthase (cGAS), detect viral nucleic acids and trigger IRF3 phosphorylation and translocation, leading to the transcription of IRF3-responsive genes such as *ISG56* and *IFN-β*.[39]

In two *HDAC5*$^{-/-}$ HeLa cell lines and two *HDAC5*$^{-/-}$ HEK293T cell lines, we show diminished activation of IRF3-dependent gene expression following SeV infection; conversely, overexpression of HDAC5 enhanced pathway activation. Interestingly, overexpression of HDAC5 alone was not stimulatory under the conditions tested, but HDAC5 augmented activation induced by SeV infection or co-expression of TRIFΔRIP. Mechanistically, the HDAC activity of HDAC5 was not required for pathway stimulation because re-introduction of catalytically inactive HDAC5 H893F into *HDAC5*$^{-/-}$ cells restored IRF3 signaling as well as WT HDAC5. Another class IIa HDAC, HDAC4, and HDAC1 were unable to complement the loss of HDAC5, showing that the defect is specific to HDAC5.

The fact that HDAC5 is not stimulatory alone but augments other activating signals is similar to the situation with Spir-1, an actin-nucleating protein that has been reported recently to augment IRF3 activation downstream of MAVS- and RIG-I-mediated RNA sensing but alone was not stimulatory.[40] Both HDAC5 and Spir-1 are targeted by specific VACV proteins. In the case of Spir-1, the viral antagonist is protein K7,[40] a Bcl-2 family member[41] that is made early during infection, contributes to virulence,[42] and antagonizes the IRF3 pathway by interacting directly with both Spir-1 and DDX3 and so also antagonizing IRF3 activation at the level of TRAF family member-associated NF-κB activator (TANK) binding kinase 1 (TBK-1).[43] K7 seems to functionally impair its cellular targets without inducing their degradation. In the case of HDAC5, the viral antagonist is protein C6,[12] another small intracellular protein that is expressed early during infection and contributes to virus virulence[37] and is also predicted to be a member of the VACV Bcl-2 family.[34] Like K7, protein C6 is also multifunctional and inhibits IRF3 activation in more than one way. Previously, it has been shown to co-precipitate with SINTBAD, TANK, and NAP1 and diminish IRF3/IRF7 activation at the level of TBK-1[37] and is shown here to bind directly to HDAC5 to antagonize TRIF-mediated IRF3 activation. Whereas K7 does not induce degradation of cellular binding partners Spir-1 and DDX3, C6 is able to induce the proteasome-dependent degradation of HDAC4,[11] HDAC5,[12] and TRIM5α.[13]

Previous proteomic analysis showed that HDAC5 co-purifies with several members of the D-E-A-D (DEAD) box protein (DDX) family, including DDX1, DDX17, DDX20, DDX3x, and DDX47.[44] These proteins sense cytosolic RNA, activate innate immune pathways, and restrict virus infection.[45–49] Given these findings, HDAC5 might play a critical role in restricting orthopoxvirus infection by regulating the TRIF-activated IRF3 pathway via DDX proteins. The precise mechanism by which HDAC5

augments IRF3 activation remains to be determined in a future study, but the demonstration that HDAC5 does not enhance activation induced by RIG-I-CARD or MAVS but does enhance TRIF-ΔRIP-induced activation identifies its approximate site of action, which is different from that of Spir-1.[40] Overall, this additional role of HDAC5 adds to the list of other activities of this multifunctional protein and contributes to knowledge of cellular proteins regulating the IRF3 pathway.

The interaction between VACV protein C6 and HDAC5 has been shown to be direct, and the region needed was mapped to HDAC5 residues 67–171 which includes the highly conserved glutamine-rich domain needed for homodimerization.[24] The HDAC5 interaction site was predicted more precisely by AFM to be aa residues 92–118. This region includes F98, which is conserved in HDAC5s from different mammals that can be infected with orthopoxviruses and is required for homodimerization.[24] Mutagenesis of this residue (F98R) prevented interaction with C6. C6 is highly conserved in orthopoxviruses, and residues F72 and F75 were predicted by AFM to interact with HDAC5, a prediction investigated by mutagenesis. C6 interacts with the region of HDAC5 needed for homodimerization, and, consequently, C6 blocks dimerization. C6 also blocked HDAC5 heterodimerization with HDAC4. While the exact molecular mechanism of the HDAC4-HDAC5 interaction is unclear, our study suggests that the interaction between HDAC4 and HDAC5 is mediated by F98 on HDAC5. The functional consequences of C6 disrupting HDAC5 homodimerization or heterodimerization with HDAC4 remain to be determined. Like its interaction with HDAC5, C6 interacts with HDAC4 directly, but mutagenesis of F93, the counterpart of F98 on HDAC5, did not abolish the interaction between HDAC4 and C6, suggesting the presence of an additional interaction site in the N-terminal domain of HDAC4.

C6 is the only VACV protein needed for HDAC5 degradation because a VACV strain lacking C6 does not degrade HDAC5, and a cell line that expresses C6 inducibly causes degradation outwith infection. Degradation is prevented by MG132 and is therefore proteasome dependent. Since C6 has a predicted Bcl-2 fold and lacks motifs found in other ubiquitin ligases, its presumed mechanism of action is via recruitment of one or more cellular E3 ligase to ubiquitylate its cellular targets. The same mechanism has been proposed for degradation of HDAC4[11] and TRIM5α,[13] although the E3 ligase(s) remain(s) unknown. C6 is highly conserved in orthopoxviruses, and C6 orthologs from VACV, RPXV, CPXV-BR, CMLV, MPXV, and VARV all degrade HDAC5 outwith infection. Infection with three different VACV strains, as well as CPXV-BR, RPXV, CMVL, CPXV-E, and MPXV, all induce HDAC5 degradation. Furthermore, HDAC5 is shown to restrict the replication of several orthopoxviruses, including VACV, CPXV-BR, CPXV-E, RPXV, and CMLV.

C6 residues F72 and F75 were predicted by AFM to mediate interaction with HDAC5 and mutagenesis or either residue prevented interaction with HDAC5 while not affecting the coIP with other C6 partners, such as STAT2[38] and SINTBAD.[37] These mutations also prevented C6 causing redistribution of HDAC5 to the cytoplasm and diminished its inhibition of IRF3 activation. Residual inhibitory activity is likely attributable to C6's ability to co-immunoprecipitate with SINTBAD, NAP1, and TANK scaffold proteins regulating IRF3 at the level of TBK-1.

In this study, we showed that C6 also interacts with HDAC4 via its N-terminal domain, a conserved domain that present in HDAC5 and HDAC9. Given the structural similarity between HDAC4, HDAC5, and HDAC9, we hypothesize that HDAC9 may also be targeted by VACV for proteasome-dependent degradation, mediated by C6. Although HDAC9 was not identified in our previous temporal proteomics analysis due to the lack of unique peptides and its low abundance in HFFFs, further investigation could elucidate the role of HDAC9 in VACV infection and innate immune pathways. Specifically, additional cell lines expressing higher levels of HDAC9 are needed to examine its abundance and function during VACV infection.

HDAC5 is overexpressed in several cancers, including colorectal, breast, and lung cancer and neuroblastoma, and silencing of HDAC5 has been shown to reduce cancer cell motility and invasion.[50,51] HDAC inhibitors (HDACis) are a promising therapy for various diseases, including neurodegenerative and inflammatory disorders as well as cancer.[52–54] Despite extensive studies, the development of selective inhibitors targeting individual HDAC subfamilies remains a challenge due to the conservation of the enzymatic domain. In this study, we investigated the mechanism of C6 and HDAC5 interaction and found that it degrades and interacts directly with HDAC5 through a conserved phenylalanine residue present in other class IIa HDACs (HDAC4, HDAC5, and HDAC9) and HDAC5 proteins from different species. These findings suggest a potential avenue for the development of specific inhibitors targeting class IIa HDACs for therapeutic use.

In summary, this study provides evidence that HDAC5 is important for the activation of the TRIF-activated IRF3 pathway and is targeted by various orthopoxviruses for proteasome-dependent degradation. C6 interacts directly with HDAC5 via three phenylalanine residues, and mutagenesis at the phenylalanine on either HDAC5 or C6 abrogated the interaction, confirming the predicted co-structure. The study also reveals that C6-mediated immune evasion is partly achieved through targeting HDAC5, as C6 mutants that cannot interact with HDAC5 are less efficient at suppressing the activation of the IRF3 pathway compared with WT C6.

Limitations of this study

This study demonstrates that HDAC5 is targeted for degradation by the C6 protein from many orthopoxviruses and that HDAC5 enhances activation of IRF3 driven by some stimuli, such as SeV infection or TRIF overexpression. However, it does not affect activation by RIG-I or MAVS. A limitation of the study is that the detailed mechanism by which HDAC5 enhances IRF3 activation remains to be determined.

STAR★METHODS

Detailed methods are provided in the online version of this paper and include the following:

- KEY RESOURCES TABLE
- RESOURCE AVAILABILITY

- ○ Lead contact
- ○ Materials availability
- ○ Data and code availability
- ● EXPERIMENTAL MODEL AND STUDY PARTICIPANT DETAILS
  - ○ Materials
  - ○ Plasmids and viruses
  - ○ Antibodies and reagents
- ● METHOD DETAILS
  - ○ Virus infection and titration
  - ○ Viral DNA preparation
  - ○ Lentivirus preparation and transduction
  - ○ T-REx 293 cell line construction
  - ○ Immunofluorescence
  - ○ Reporter gene assay
  - ○ Affinity purification
  - ○ Recombinant protein expression and purification
  - ○ *In vitro* expression system
  - ○ GSH beads pull-down of GST-C6 and GST
  - ○ Prediction of the C6-HDAC5 co-structure by AlphaFold2
- ● QUANTIFICATION AND STATISTICAL ANALYSIS

SUPPLEMENTAL INFORMATION

Supplemental information can be found online at https://doi.org/10.1016/j.celrep.2024.113788.

ACKNOWLEDGMENTS

We thank the members of Smith's lab for helpful discussions. We would also like to thank Prof. Emma Thomson and Dr. Chris Davis from MRC-University of Glasgow Centre for Virus Research for providing the MPXV-CVR-S1 C6 sequence. This work was supported by an MRC grant (MR/W025590/1), a Wellcome Trust Principal Research Fellowship (090315), a BBSRC grant (BB/X011542/1), the Isaac Newton Trust, and the University of Cambridge. Y.Z. was supported by a PhD studentship from the Department of Pathology, University of Cambridge. S.S. and J.M. were supported by the Department of Pathology, University of Cambridge. A.S. was supported by a summer studentship from the Lister Institute of Preventive Medicine.

AUTHOR CONTRIBUTIONS

Y.L., Y.Z., C.G., and G.L.S. designed the experiments. Y.L., Y.Z., C.G., S.S., J.M., and A.S. performed the experiments. Y.L., Y.Z., C.G., S.S., J.M., and A.S. analyzed the data. Y.L. and G.L.S. wrote and edited the manuscript. G.L.S. and Y.L. supervised the project. G.L.S. acquired funding.

DECLARATION OF INTERESTS

The authors declare no competing interests.

Received: September 18, 2023
Revised: December 18, 2023
Accepted: January 26, 2024
Published: March 10, 2024

REFERENCES

1. Moss, B., and Smith, G.L. (2021). Poxviridae: the viruses and their replication. In Fields Virology: DNA Viruses, P.M. Howley and D.M. Knipe, eds. (Wolters Kluwer Inc), pp. 573–613.
2. Fenner, F., Henderson, D.A., Arita, I., Jezek, Z., and Ladnyi, I.D. (1988). Smallpox and its Eradication.
3. Mackett, M., Smith, G.L., and Moss, B. (1982). Vaccinia virus: a selectable eukaryotic cloning and expression vector. Proc. Natl. Acad. Sci. USA *79*, 7415–7419. https://doi.org/10.1073/pnas.79.23.7415.
4. Panicali, D., and Paoletti, E. (1982). Construction of poxviruses as cloning vectors: insertion of the thymidine kinase gene from herpes simplex virus into the DNA of infectious vaccinia virus. Proc. Natl. Acad. Sci. USA *79*, 4927–4931. https://doi.org/10.1073/pnas.79.16.4927.
5. Panicali, D., Davis, S.W., Weinberg, R.L., and Paoletti, E. (1983). Construction of live vaccines by using genetically engineered poxviruses: biological activity of recombinant vaccinia virus expressing influenza virus hemagglutinin. Proc. Natl. Acad. Sci. USA *80*, 5364–5368. https://doi.org/10.1073/pnas.80.17.5364.
6. Smith, G.L., Mackett, M., and Moss, B. (1983). Infectious vaccinia virus recombinants that express hepatitis B virus surface antigen. Nature *302*, 490–495. https://doi.org/10.1038/302490a0.
7. Smith, G.L., Murphy, B.R., and Moss, B. (1983). Construction and characterization of an infectious vaccinia virus recombinant that expresses the influenza hemagglutinin gene and induces resistance to influenza virus infection in hamsters. Proc. Natl. Acad. Sci. USA. *80*, 7155–7159. https://doi.org/10.1073/pnas.80.23.7155.
8. Heo, J., Reid, T., Ruo, L., Breitbach, C.J., Rose, S., Bloomston, M., Cho, M., Lim, H.Y., Chung, H.C., Kim, C.W., et al. (2013). Randomized dose-finding clinical trial of oncolytic immunotherapeutic vaccinia JX-594 in liver cancer. Nat. Med. *19*, 329–336. https://doi.org/10.1038/nm.3089.
9. Kirn, D.H., and Thorne, S.H. (2009). Targeted and armed oncolytic poxviruses: a novel multi-mechanistic therapeutic class for cancer. Nat. Rev. Cancer *9*, 64–71. https://doi.org/10.1038/nrc2545.
10. Smith, G.L., Benfield, C.T.O., Maluquer de Motes, C., Mazzon, M., Ember, S.W.J., Ferguson, B.J., and Sumner, R.P. (2013). Vaccinia virus immune evasion: mechanisms, virulence and immunogenicity. J. Gen. Virol. *94*, 2367–2392. https://doi.org/10.1099/vir.0.055921-0.
11. Lu, Y., Stuart, J.H., Talbot-Cooper, C., Agrawal-Singh, S., Huntly, B., Smid, A.I., Snowden, J.S., Dupont, L., and Smith, G.L. (2019). Histone deacetylase 4 promotes type I interferon signaling, restricts DNA viruses, and is degraded via vaccinia virus protein C6. Proc. Natl. Acad. Sci. USA *116*, 11997–12006. https://doi.org/10.1073/pnas.1816399116.
12. Soday, L., Lu, Y., Albarnaz, J.D., Davies, C.T.R., Antrobus, R., Smith, G.L., and Weekes, M.P. (2019). Quantitative temporal proteomic analysis of vaccinia virus infection reveals regulation of histone deacetylases by an interferon antagonist. Cell Rep. *27*, 1920–1933.e7. https://doi.org/10.1016/j.celrep.2019.04.042.
13. Zhao, Y., Lu, Y., Richardson, S., Sreekumar, M., Albarnaz, J.D., and Smith, G.L. (2023). TRIM5α restricts poxviruses and is antagonised by CypA and the viral protein C6. Nature *620*, 873–880. https://www.nature.com/articles/s41586-023-06401-0.
14. Hu, Y., and Suliman, B.A. (2017). Roles of HDACs in the responses of innate immune cells and as targets in inflammatory diseases. Adv. Exp. Med. Biol. *1024*, 91–110. https://doi.org/10.1007/978-981-10-5987-2_4.
15. Herbein, G., and Wendling, D. (2010). Histone deacetylases in viral infections. Clin. Epigenetics *1*, 13–24. https://doi.org/10.1007/s13148-010-0003-5.
16. Cao, J., Sun, L., Aramsangtienchai, P., Spiegelman, N.A., Zhang, X., Huang, W., Seto, E., and Lin, H. (2019). HDAC11 regulates type I interferon signaling through defatty-acylation of SHMT2. Proc. Natl. Acad. Sci. USA *116*, 5487–5492. https://doi.org/10.1073/pnas.1815365116.
17. Yang, Q., Tang, J., Pei, R., Gao, X., Guo, J., Xu, C., Wang, Y., Wang, Q., Wu, C., Zhou, Y., et al. (2019). Host HDAC4 regulates the antiviral response by inhibiting the phosphorylation of IRF3. J. Mol. Cell Biol. *11*, 158–169. https://doi.org/10.1093/jmcb/mjy035.
18. Xu, C., Tang, J., Yang, Q., Zhao, H., Liu, Y., Cao, J., Zhou, Y., Chen, X., and Chen, J. (2021). Histone deacetylase 5 deacetylates the phosphatase

CellPress
OPEN ACCESS

PP2A for positively regulating NF-kappaB signaling. J. Biol. Chem. *297*, 101380. https://doi.org/10.1016/j.jbc.2021.101380.

19. Yang, L., Chen, S., Zhao, Q., Pan, C., Peng, L., Han, Y., Li, L., Ruan, J., Xia, J., Yang, H., et al. (2022). Histone deacetylase 3 contributes to the antiviral innate immunity of macrophages by interacting with FOXK1 to regulate STAT1/2 transcription. Cell Rep. *38*, 110302. https://doi.org/10.1016/j.celrep.2022.110302.

20. Kumar, V., Kundu, S., Singh, A., and Singh, S. (2022). Understanding the role of histone deacetylase and their inhibitors in neurodegenerative disorders: current targets and future perspective. Curr. Neuropharmacol. *20*, 158–178. https://doi.org/10.2174/1570159X19666210609160017.

21. Bagchi, R.A., and Weeks, K.L. (2019). Histone deacetylases in cardiovascular and metabolic diseases. J. Mol. Cell. Cardiol. *130*, 151–159. https://doi.org/10.1016/j.yjmcc.2019.04.003.

22. Li, Y., and Seto, E. (2016). HDACs and HDAC inhibitors in cancer development and therapy. Cold Spring Harb. Perspect. Med. *6*, a026831. https://doi.org/10.1101/cshperspect.a026831.

23. Haberland, M., Montgomery, R.L., and Olson, E.N. (2009). The many roles of histone deacetylases in development and physiology: implications for disease and therapy. Nat. Rev. Genet. *10*, 32–42. https://doi.org/10.1038/nrg2485.

24. Guo, L., Han, A., Bates, D.L., Cao, J., and Chen, L. (2007). Crystal structure of a conserved N-terminal domain of histone deacetylase 4 reveals functional insights into glutamine-rich domains. Proc. Natl. Acad. Sci. USA *104*, 4297–4302. https://doi.org/10.1073/pnas.0608041104.

25. Lomonte, P., Thomas, J., Texier, P., Caron, C., Khochbin, S., and Epstein, A.L. (2004). Functional interaction between class II histone deacetylases and ICP0 of herpes simplex virus type 1. J. Virol. *78*, 6744–6757. https://doi.org/10.1128/JVI.78.13.6744-6757.2004.

26. Lee, H.R., Li, F., Choi, U.Y., Yu, H.R., Aldrovandi, G.M., Feng, P., Gao, S.J., Hong, Y.K., and Jung, J.U. (2018). Deregulation of HDAC5 by viral interferon regulatory factor 3 plays an essential role in Kaposi's sarcoma-associated herpesvirus-induced lymphangiogenesis. mBio *9*, e02217–17. https://doi.org/10.1128/mBio.02217-17.

27. Portal, D., Rosendorff, A., and Kieff, E. (2006). Epstein-Barr nuclear antigen leader protein coactivates transcription through interaction with histone deacetylase 4. Proc. Natl. Acad. Sci. USA *103*, 19278–19283. https://doi.org/10.1073/pnas.0609320103.

28. Taha, T.Y., Anirudhan, V., Limothai, U., Loeb, D.D., Petukhov, P.A., and McLachlan, A. (2020). Modulation of hepatitis B virus pregenomic RNA stability and splicing by histone deacetylase 5 enhances viral biosynthesis. PLoS Pathog. *16*, e1008802. https://doi.org/10.1371/journal.ppat.1008802.

29. Downes, M., Ordentlich, P., Kao, H.Y., Alvarez, J.G., and Evans, R.M. (2000). Identification of a nuclear domain with deacetylase activity. Proc. Natl. Acad. Sci. USA *97*, 10330–10335. https://doi.org/10.1073/pnas.97.19.10330.

30. Cooray, S., Bahar, M.W., Abrescia, N.G.A., McVey, C.E., Bartlett, N.W., Chen, R.A.J., Stuart, D.I., Grimes, J.M., and Smith, G.L. (2007). Functional and structural studies of the vaccinia virus virulence factor N1 reveal a Bcl-2-like anti-apoptotic protein. J. Gen. Virol. *88*, 1656–1666, 88/6/1656 [pii]. https://doi.org/10.1099/vir.0.82772-0.

31. Maluquer de Motes, C., Cooray, S., Ren, H., Almeida, G.M.F., McGourty, K., Bahar, M.W., Stuart, D.I., Grimes, J.M., Graham, S.C., and Smith, G.L. (2011). Inhibition of apoptosis and NF-kappaB activation by vaccinia protein N1 occur via distinct binding surfaces and make different contributions to virulence. PLoS Pathog. *7*, e1002430. https://doi.org/10.1371/journal.ppat.1002430.

32. Gloeckner, C.J., Boldt, K., Schumacher, A., Roepman, R., and Ueffing, M. (2007). A novel tandem affinity purification strategy for the efficient isolation and characterisation of native protein complexes. Proteomics *7*, 4228–4234. https://doi.org/10.1002/pmic.200700038.

33. Park, S.Y., and Kim, J.S. (2020). A short guide to histone deacetylases including recent progress on class II enzymes. Exp. Mol. Med. *52*, 204–212. https://doi.org/10.1038/s12276-020-0382-4.

34. González, J.M., and Esteban, M. (2010). A poxvirus Bcl-2-like gene family involved in regulation of host immune response: sequence similarity and evolutionary history. Virol. J. *7*, 59. https://doi.org/10.1186/1743-422X-7-59.

35. Jumper, J., Evans, R., Pritzel, A., Green, T., Figurnov, M., Ronneberger, O., Tunyasuvunakool, K., Bates, R., Žídek, A., Potapenko, A., et al. (2021). Highly accurate protein structure prediction with AlphaFold. Nature *596*, 583–589. https://doi.org/10.1038/s41586-021-03819-2.

36. Evans, R., O'Neill, M., Pritzel, A., Antropova, N., Senior, A., Green, T., Žídek, A., Bates, R., Blackwell, R., Yim, J., et al. (2022). Protein complex prediction with alphafold-multimer. Preprint at BioRxiv. https://www.biorxiv.org/content/10.1101/2021.10.04.463034v2.

37. Unterholzner, L., Sumner, R.P., Baran, M., Ren, H., Mansur, D.S., Bourke, N.M., Randow, F., Smith, G.L., and Bowie, A.G. (2011). Vaccinia virus protein C6 is a virulence factor that binds TBK-1 adaptor proteins and inhibits activation of IRF3 and IRF7. PLoS Pathog. *7*, e1002247, D-11-00715 [pii]. https://doi.org/10.1371/journal.ppat.1002247. PPATHOGENS-.

38. Stuart, J.H., Sumner, R.P., Lu, Y., Snowden, J.S., and Smith, G.L. (2016). Vaccinia virus protein C6 inhibits type I IFN signalling in the nucleus and binds to the transactivation domain of STAT2. PLoS Pathog. *12*, e1005955. https://doi.org/10.1371/journal.ppat.1005955.

39. McNab, F., Mayer-Barber, K., Sher, A., Wack, A., and O'Garra, A. (2015). Type I interferons in infectious disease. Nat. Rev. Immunol. *15*, 87–103. https://doi.org/10.1038/nri3787.

40. Torres, A.A., Macilwee, S.L., Rashid, A., Cox, S.E., Albarnaz, J.D., Bonjardim, C.A., and Smith, G.L. (2022). The actin nucleator Spir-1 is a virus restriction factor that promotes innate immune signalling. PLoS Pathog. *18*, e1010277. https://doi.org/10.1371/journal.ppat.1010277.

41. Kalverda, A.P., Thompson, G.S., Vogel, A., Schröder, M., Bowie, A.G., Khan, A.R., and Homans, S.W. (2009). Poxvirus K7 protein adopts a Bcl-2 fold: biochemical mapping of its interactions with human DEAD box RNA helicase DDX3. J. Mol. Biol. *385*, 843–853, S0022-2836(08)01194-7 [pii]. https://doi.org/10.1016/j.jmb.2008.09.048.

42. Benfield, C.T.O., Ren, H., Lucas, S.J., Bahsoun, B., and Smith, G.L. (2013). Vaccinia virus protein K7 is a virulence factor that alters the acute immune response to infection. J. Gen. Virol. *94*, 1647–1657. https://doi.org/10.1099/vir.0.052670-0.

43. Schröder, M., Baran, M., and Bowie, A.G. (2008). Viral targeting of DEAD box protein 3 reveals its role in TBK1/IKKepsilon-mediated IRF activation. The EMBO journal *27*, 2147–2157. https://doi.org/10.1038/emboj.2008.143.

44. Greco, T.M., Yu, F., Guise, A.J., and Cristea, I.M. (2011). Nuclear import of histone deacetylase 5 by requisite nuclear localization signal phosphorylation. Mol. Cell. Proteomics *10*, M110.004317. https://doi.org/10.1074/mcp.M110.004317.

45. Zhang, Z., Kim, T., Bao, M., Facchinetti, V., Jung, S.Y., Ghaffari, A.A., Qin, J., Cheng, G., and Liu, Y.J. (2011). DDX1, DDX21, and DHX36 helicases form a complex with the adaptor molecule TRIF to sense dsRNA in dendritic cells. Immunity *34*, 866–878. https://doi.org/10.1016/j.immuni.2011.03.027.

46. Taschuk, F., and Cherry, S. (2020). DEAD-box helicases: sensors, regulators, and effectors for antiviral defense. Viruses *12*. https://doi.org/10.3390/v12020181.

47. Greenwood, E.J.D., Williamson, J.C., Sienkiewicz, A., Naamati, A., Matheson, N.J., and Lehner, P.J. (2019). Promiscuous targeting of cellular proteins by Vpr drives systems-level proteomic remodeling in HIV-1 infection. Cell Rep. *27*, 1579–1596.e7. https://doi.org/10.1016/j.celrep.2019.04.025.

48. Pallett, M.A., Lu, Y., and Smith, G.L. (2022). DDX50 is a viral restriction factor that enhances IRF3 activation. Viruses *14*, 316. https://doi.org/10.3390/v14020316.

49. Khadivjam, B., Bonneil, É., Thibault, P., and Lippé, R. (2023). RNA helicase DDX3X modulates herpes simplex virus 1 nuclear egress. Commun. Biol. *6*, 134. https://doi.org/10.1038/s42003-023-04522-w.

50. He, P., Liang, J., Shao, T., Guo, Y., Hou, Y., and Li, Y. (2015). HDAC5 promotes colorectal cancer cell proliferation by up-regulating DLL4 expression. Int. J. Clin. Exp. Med. *8*, 6510–6516.

51. Yang, J., Gong, C., Ke, Q., Fang, Z., Chen, X., Ye, M., and Xu, X. (2021). Insights into the function and clinical application of HDAC5 in cancer management. Front. Oncol. *11*, 661620. https://doi.org/10.3389/fonc.2021.661620.

52. Shukla, S., and Tekwani, B.L. (2020). Histone deacetylases inhibitors in neurodegenerative diseases, neuroprotection and neuronal differentiation. Front. Pharmacol. *11*, 537. https://doi.org/10.3389/fphar.2020.00537.

53. Hull, E.E., Montgomery, M.R., and Leyva, K.J. (2016). HDAC inhibitors as epigenetic regulators of the immune system: Impacts on cancer therapy and inflammatory diseases. BioMed Res. Int. *2016*, 8797206. https://doi.org/10.1155/2016/8797206.

54. Eckschlager, T., Plch, J., Stiborova, M., and Hrabeta, J. (2017). Histone deacetylase inhibitors as anticancer drugs. Int. J. Mol. Sci. *18*, 1414. https://doi.org/10.3390/ijms18071414.

55. Parkinson, J.E., and Smith, G.L. (1994). Vaccinia virus gene A36R encodes a Mr 43-50 K protein on the surface of extracellular enveloped virus. Virology *204*, 376–390. https://doi.org/10.1006/viro.1994.1542.

56. Alcamí, A., and Smith, G.L. (1995). Vaccinia, cowpox, and camelpox viruses encode soluble gamma interferon receptors with novel broad species specificity. J. Virol. *69*, 4633–4639. https://doi.org/10.1128/JVI.69.8.4633-4639.1995.

57. Maluquer de Motes, C., Schiffner, T., Sumner, R.P., and Smith, G.L. (2014). Vaccinia virus virulence factor N1 can be ubiquitylated on multiple lysine residues. J. Gen. Virol. *95*, 2038–2049. https://doi.org/10.1099/vir.0.065664-0.

58. Lu, Y., Orr, A., and Everett, R.D. (2016). Stimulation of the replication of ICP0-null mutant herpes simplex virus 1 and pp71-deficient human cytomegalovirus by Epstein-Barr virus tegument protein BNRF1. J. Virol. *90*, 9664–9673. https://doi.org/10.1128/JVI.01224-16.

59. Carter, G.C., Rodger, G., Murphy, B.J., Law, M., Krauss, O., Hollinshead, M., and Smith, G.L. (2003). Vaccinia virus cores are transported on microtubules. J. Gen. Virol. *84*, 2443–2458. https://doi.org/10.1099/vir.0.19271-0.

STAR★METHODS

KEY RESOURCES TABLE

REAGENT or RESOURCE	SOURCE	IDENTIFIER
Antibodies		
Mouse anti-HDAC5	SANTA CRUZ	Cat# Sc133106; RRID:AB_2116793
Mouse anti-α-tubulin	SANTA CRUZ	Cat# Sc-69970; RRID:AB_2303941
Mouse anti-HA	BioLegend	Cat# 901501; RRID:AB_2565006
Mouse anti-D8	Laboratory of Geoffrey L Smith	Parkinson and Smith, 1994[55]
Rabbit anti-FLAG	Sigma-Aldrich	Cat# F7425; RRID:AB_439687
Rabbit anti-phospho-IRF3 (Ser386)	Cell Signaling	Cat# 4947S; RRID:AB_823547
Rabbit anti-actin	Sigma-Aldrich	Cat# A2066; RRID:AB_476693
Rabbit anti-C6	Laboratory of Geoffrey L Smith	Unterholzner et al., 2011[37]
IRDye® 800CW Goat anti-Mouse IgG	LICOR	Cat# 926-32210: RRID:AB_621842
IRDye® 800CW Goat anti-Rabbit IgG	LICOR	Cat# 926-32211; RRID:AB_621843
IRDye® 680RD Goat anti-Rabbit IgG	LICOR	Cat# 926-68071; RRID:AB_10956166
IRDye® 680RD Goat anti-Mouse IgG	LICOR	Cat# 926-68070; RRID:AB_10956588
Alexa Fluor 546 Goat anti-Rabbit IgG (H + L)	Invitrogen	Cat# A-11035; RRID:AB_2534093
Alexa Fluor 488 Donkey anti-Mouse IgG (H + L)	Invitrogen	Cat# R37114; RRID:AB_2556542
Bacterial and virus strains		
RPXV strain Utrecht	Laboratory of Geoffrey L Smith	Alcamí et al., 1995[56]
CPXV strain Brighton Red	Laboratory of Geoffrey L Smith	Alcamí et al., 1995[56]
CPXV-E	Laboratory of Geoffrey L Smith	Alcamí et al., 1995[56]
CMLV strain CMS	Laboratory of Geoffrey L Smith	Alcamí et al., 1995[56]
MPXV_CVR	MRC-University of Glasgow Center for Virus Research	Zhao et al., 2023[13]
Sendai Virus Cantell strain	Steve Goodbourn, St George's Hospital Medical School, University of London	N/A
Chemicals, peptides, and recombinant proteins		
4′, 6-Diamidino-2-Phenylindole, Dihydrochloride (DAPI)	ThermoFisher	D1306
Opti-MEM™ reduced serum medium	ThermoFisher	31985070
TransIT-LT1 transfection reagent	Mirus	MIR 2306
TNF-α	Peprotech	300-001A
IL-1β	Peprotech	200-01A
IFN-α	Peprotech	300-02AA
IFN-γ	Peprotech	300–02
cOmplete™ Protease Inhibitor Cocktail	Roche	11697498001
phosphatase inhibitor PhosSTOP™	Roche	4906845001
ANTI-FLAG® M2 Affinity Gel	MERCK	A2220
Anti-HA-Agarose	MERCK	A2095
Strep-Tactin®XT 4Flow® resin	iba	2-5010-002
Glutathione Sepharose 4B GST-tagged protein purification resin	Cytiva	17075605
MG132	Sigma-Aldrich	SML1135
DMEM	ThermoFisher	41966029
10 x MEM	ThermoFisher	11430030
Opti-MEM™ I Reduced Serum Medium	ThermoFisher	31985070

(*Continued on next page*)

Continued

REAGENT or RESOURCE	SOURCE	IDENTIFIER
Sodium Bicarbonate 7.5% solution	ThermoFisher	25080094
L-Glutamine	ThermoFisher	25030–081
FBS	PAN Biotech UK	P40-37500HI
Penicillin-Streptomycin	ThermoFisher	15140122
Puromycin (solution)	InvivoGen	ant-pr-1
Paraformaldehyde 16% Aqueous Solution, EM Grade	Electron Microscopy Sciences	15711
Coelenterazine (Renilla Luciferase substrate)	Nanolight Technology	303
D-Luciferine	Nanolight Technology	306
Co-enzyme A	Nanolight Technology	309
Fast SYBR™ Green Master Mix	ThermoFisher	4385612
RNaseOUT™ Recombinant Ribonuclease Inhibitor	ThermoFisher	10777019
SuperScript™ III Reverse Transcriptase	ThermoFisher	18080093
Oligo(dT)20 Primer Invitrogen™	ThermoFisher	18418020
dNTP	ThermoFisher	R0181
Passive Lysis 5X Buffer	Promega	E1941
Critical commercial assays		
Plasmid DNA sequencing	SourceBioscience	N/A
Experimental models: Cell lines		
HeLa (human cervical adenocarcinoma epithelial cell line)	ATCC	CCL-2
H5KO1	Laboratory of Geoffrey L Smith	Soday et al., 2019[12]
H5KO2	Laboratory of Geoffrey L Smith	Soday et al., 2019[12]
HEK-293T (human embryo kidney epithelial cell line)	ATCC	CRL-11268
H5KO4	Laboratory of Geoffrey L Smith	Soday et al., 2019[12]
H5KO3	Laboratory of Geoffrey L Smith	Soday et al., 2019[12]
T-REx-293	Life Technologies	R71007
HFFF	Laboratory of Michael Weekes	Soday et al., 2019[12]
RK13	ATCC	CCL-37
BSC-1	ATCC	CCL-26
U2OS	ATCC	HTB-96™
U2OS.TetO.TetR.EYFP	This paper	N/A
U2OS.TetO.TetR.HDAC5	This paper	N/A
Oligonucleotides		
*ISG56*_FWD ACCATGGGAGAGAATGCTGAT	Laboratory of Geoffrey L Smith	Pallett et al., 2022[48]
*ISG56*_REV GCCAGGAGGTTGTGC	Laboratory of Geoffrey L Smith	Pallett et al., 2022[48]
*IFN-β*_FWD CATCAACTATAAGCAGCTCCA	Laboratory of Geoffrey L Smith	Pallett et al., 2022[48]
*IFN-β*_REV TTCAAGTGGAGAGCAGTTGAG	Laboratory of Geoffrey L Smith	Pallett et al., 2022[48]
*GAPDH*_FWD ATCAACGACCCCTTCATTGACC	Laboratory of Geoffrey L Smith	Pallett et al., 2022[48]
*GAPDH*_REV CCAGTAGACTCCACGACATACTCAGC	Laboratory of Geoffrey L Smith	Pallett et al., 2022[48]

(*Continued on next page*)

CellPress
OPEN ACCESS

Continued

REAGENT or RESOURCE	SOURCE	IDENTIFIER
Recombinant DNA		
pcDNA3.HDAC5-FLAG	Addgene	13822
pcDNA3.HDAC4-FLAG	Addgene	13821
pcDNA3.HDAC1-FLAG	Addgene	13820
pF3A	Promega	L5671
pcDNA3.HAcoC6	Laboratory of Geoffrey L Smith	Maluquer de Motes et al., 2014[57]
pcDNA3.N1-HA	Laboratory of Geoffrey L Smith	Maluquer de Motes et al., 2014[57]
pcDNA3.TAP-codon optimized C6 (TAPcoC6)	Laboratory of Geoffrey L Smith	Maluquer de Motes et al., 2014[57]
pcDNA3.N1-TAP	Laboratory of Geoffrey L Smith	Maluquer de Motes et al., 2011[57]
pcDNA4-TAP-N1	Laboratory of Geoffrey L Smith	Maluquer de Motes et al., 2011[57]
pcDNA3.TAPcoC6_F72R	This paper	N/A
pcDNA3.TAPcoC6_F75R	This paper	N/A
pcDNA3.HAcoC6_F72R	This paper	N/A
pcDNA3.HAcoC6_F75R	This paper	N/A
pcDNA3.HDAC5N-FLAG (HDAC5 1–684)	This paper	N/A
pcDNA3.HDAC5C-FLAG (HDAC5 684–1122)	This paper	N/A
pcDNA3.HDAC4N-FLAG (HDAC4 1–650)	This paper	N/A
pcDNA3.HDAC4C-FLAG (HDAC4 651–1084)	This paper	N/A
pcDNA3.HDAC5_H893F	This paper	N/A
pcDNA3.HDAC5N_F95R-FLAG	This paper	N/A
pcDNA3.HDAC5N_F98R-FLAG	This paper	N/A
pcDNA3.HDAC5N_F95R-HA	This paper	N/A
pcDNA3.HDAC5N_F98R-HA	This paper	N/A
pcDNA4.TAP-C6-VACV	This paper	N/A
pcDNA4.TAP-C6-RPXV	This paper	N/A
pcDNA4.TAP-C6-CPXV-BR	This paper	N/A
pcDNA4.TAP-C6-CPXV-E	This paper	N/A
pcDNA4.TAP-C6-CMLV	This paper	N/A
pcDNA4.TAP-C6-MPXV-Zaire	This paper	N/A
pcDNA4.TAP-C6-MPXV-CVR-S1	This paper	N/A
pcDNA4.TAPcoC6-VARV	This paper	N/A
pcDNA4.TAP-C6-F72R	This paper	N/A
pcDNA4.TAP-C6-F75R	This paper	N/A
pF3A.HDAC5-HA	This paper	N/A
pF3A.HDAC5N-HA	This paper	N/A
pF3A.HDAC1-HA	This paper	N/A
pF3A.TAPcoC6	This paper	N/A
pF3A.TAP-N1	This paper	N/A
pF3A.m.yc-HDAC5N	This paper	N/A
pF3A.m.yc-HDAC5ND1	This paper	N/A
pF3A.m.yc-HDAC5ND2	This paper	N/A
pF3A.m.yc-HDAC5ND3	This paper	N/A
pF3A.m.yc-HDAC4N	This paper	N/A
pF3A.m.yc-HDAC4ND1	This paper	N/A
pF3A.m.yc-HDAC4ND2	This paper	N/A

(*Continued on next page*)

Continued

REAGENT or RESOURCE	SOURCE	IDENTIFIER
pF3A.m.yc-HDAC4ND3	This paper	N/A
pOPT3G.C6	This paper	N/A
ISRE-Luc	Laboratory of Andrew Bowie	N/A
NF-κB-Luc	Laboratory of Andrew Bowie	N/A
GAS-Luc	Laboratory of Andrew Bowie	N/A
TK-*Renilla*-Luc	Laboratory of Andrew Bowie	N/A
ISG56.1-Luc	Laboratory of Ganes C Sen	N/A
pLKO.dCMV.TetO.TetR.EYFP	Laboratory of Roger Everett	Lu et al., 2016[58]
pLKO.dCMV.TetO.TetR.HDAC5-FLAG	This paper	N/A
pCMV.dR8.91 expressing signal for Lentivirus packaging	Laboratory of Heike Laman	N/A
pMD-G expressing the vesicular stomatitis virus envelope protein G	Laboratory of Heike Laman	N/A
Software and algorithms		
Image Studio™ Acquisition Software	LI-COR	Version 5.2
ImageJ	Fiji	Version 1.53
Clustal Omega	European Bioinformatics Institute	Version 1.20
Prism	GraphPad	Version 7.04
FLUOstar Omega Reader Control Software	BMG Labtech	Version 1.20
MARS Data Analysis Software	BMG Labtech	Version 2.00
ZEN Microscope Software	ZEISS	Version 6.0.0.485
ZEN Lite Microscope Software	ZEISS	Version 2.5.75.0
QuantStudio™ Real-Time software	ThermoFisher	Version 1.3

RESOURCE AVAILABILITY

Lead contact

Further information and requests for resources and reagents should be directed to and will be fulfilled by the lead contact, Geoffrey L. Smith (geoffrey.smith@path.ox.ac.uk).

Materials availability

Plasmids and cell lines generated in this study are available under request.

Data and code availability

- We are willing to share all of the data reported in this published paper.
- This paper does not report original code.
- Any additional information required to reanalyze the data reported in this work paper is available from the lead contact upon request.

EXPERIMENTAL MODEL AND STUDY PARTICIPANT DETAILS

Materials

Cell lines

HEK293T (ATCC, CRL-11268), T-REx 293 (Life Technologies, R71007), HeLa (ATCC, CCL-2), U2OS (Human bone osteosarcoma epithelial, ATCC, HTB-96), and BSC-1 (ATCC, CCL-26) cells were maintained in DMEM (Invitrogen, 11960044) supplemented with 10% fetal bovine serum (FBS, Pan Biotech, P30-3306) and 50 μg/mL of penicillin/streptomycin (P/S, Gibco, 15140122). MDCK (ATCC, NBL-2) and RK13 (ATCC, CCL-37) cells were maintained in MEM (Gibco, 11095080) supplemented with 10% FBS and 50 μg/mL of P/S. HDAC5$^{-/-}$ cell lines derived from HEK293T and HeLa were described.[12] Construction of viral protein expressing T-REx 293 cell lines is described in STAR Methods.

Plasmids and viruses

Plasmids used in the studies were from the following sources: pcDNA3.HDAC5-FLAG (Addgene, 13822), pcDNA3.HDAC4-FLAG (Addgene, 13821), pcDNA3.HDAC1-FLAG (Addgene, 13820), pF3A (Promega, L5671) were purchased from indicated sources. pcDNA3.HAcoC6, pcDNA3.N1-HA, pcDNA3.TAPcoC6,[57] pcDNA3.N1-TAP and pcDNA4-TAP-N1 were described.[31] pcDNA3.TAPcoC6-F72R, pcDNA4.TAP-C6-F72R, pcDNA3.TAPcoC6-F75R, pcDNA4.TAP-C6-F75R, pcDNA3.HAcoC6_F72R, pcDNA3.HAcoC6_F75R, pcDNA3.HDAC5N-FLAG (HDAC5 1–684), pcDNA3.HDAC5C-FLAG (HDAC5 684–1122), pcDNA3.HDAC4N-FLAG (HDAC4 1–650), pcDNA3.HDAC4C-FLAG (HDAC4 651–1084), pcDNA3.HDAC5_H893F, pcDNA3.HDAC5N_F95R-FLAG, pcDNA3.HDAC5N_F98R-FLAG, pcDNA3.HDAC5_F98R-HA, pcDNA4-TAP-C6-VACV, pcDNA4-TAP-C6-RPXV, pcDNA4-TAP-C6-CPXV-BR, pcDNA4-TAP-C6-CPXV-E, pcDNA4-TAP-C6-CMLV, pcDNA4-TAP-C6-MPXV-Zaire, pcDNA4-TAP-C6-MPXV-CVR-S1, pcDNA4-TAPcoC6-VARV, pF3A.HDAC5-HA, pF3A.HDAC5N-HA, pF3A.HDAC1-HA, pF3A.TAPcoC6, pF3A.TAP-N1, pF3A.m.yc-H5N, pF3A.m.yc-H5ND1, pF3A.m.yc-H5ND2, pF3A.m.yc-H5ND3, pF3A.m.yc-H4N, pF3A.m.yc-H4ND1, pF3A.m.yc-H4ND2, pF3A.m.yc-H4ND3 and pOPT3G-C6 plasmids were constructed for this study. Oligonucleotides used for cloning in this study are listed in Table S1. The reporter plasmids containing either ISRE, GAS, or NF-κB responsive promoters driving expression of firefly luciferase (ISRE-Luc, NF-κB-Luc, or GAS-Luc), and the transfection control plasmid with the thymidine kinase promoter driving expression of renilla luciferase (TK-*Renilla*-Luc) were gifts from Andrew Bowie, Trinity College, Dublin. ISG56.1-Luc plasmid was a gift from Ganes C. Sen, Cleveland Clinic, Cleveland, OH. Doxycycline inducible lentivirus vector pLKO.dCMV.TetO.TetR.EYFP (enhanced yellow fluorescent protein) was kindly provided by Roger Everett, MRC, Centre for Virus Research, University of Glasgow, Glasgow, UK.[58] The vector includes tetracycline operator (TetO) upstream of EYFP and T7 promoter upstream of tetracycline repressor (TetR) that creates Tet-On protein expression system with a single vector. HDAC5 with an FLAG epitope at the C-terminal end were sub-cloned into pLKO.dCMV.TetO.TetR.EYFP replacing EYFP. Plasmids pCMV.dR8.91 (expressing signal for Lentivirus packaging) and pMD-G (expressing the vesicular stomatitis virus envelope protein G) were gifts from Heike Laman (University of Cambridge, UK). Lentivirus transduction was described.[11]

All primers were ordered from Sigma-Aldrich and listed in (key resources table). Polymerase chain reactions (PCR) for cloning were performed with Q5 High-Fidelity DNA Polymerase (NEB, M0491L). Colony PCR for detection of the cloned vectors were performed with OneTaq Quick-Load 2X Master Mix with Standard Buffer (NEB, M0486L). Plasmid preparations were performed with GeneJET Plasmid Miniprep Kit (Thermo, K0503) and QIAGEN Plasmid Midi Kit (QIAGEN, 12143).

Sendai virus Cantell strain (Licence No. ITIMP17.0612A) was provided by Steve Goodbourn, St George's Hospital Medical School, University of London. VACV Western Reserve (WR) strain, Lister strain, Copenhagen strain, RPXV, CPXV-BR, CPXV-E and CMLV were described.[56] VACV WR derivative strains lacking gene *C6L* expression,[37] expressing TAP-tagged N1 or C6[57] or expressing A5-GFP fusion protein (A5GFP VACV,[59]) were described elsewhere. MPXV-CVR-S1 was isolated by MRC-University of Glasgow Center for Virus Research in Glasgow.

Antibodies and reagents

Antibodies used in this study were from the following sources: Mouse (Ms) anti-HDAC5 (SANTA CRUZ, sc-133106), Ms anti-HA (BioLegend, 901502), Ms anti-α-tubulin (SANTA CRUZ, sc-69970), Rabbit (Rb) anti-FLAG (Sigma-Aldrich, F7425), Rb anti-actin (Sigma, A2066), Alexa Fluor 546 goat anti-Rb IgG (H + L) (Invitrogen, A-11035) or Alexa Fluor 488 donkey anti-Ms IgG (H + L) (Invitrogen, R37114). DNA was stained with 4′, 6-Diamidino-2-Phenylindole, Dihydrochloride (DAPI, ThermoFisher, D1306) in IF imaging.

Plasmids were transfected using TransIT-LT1 transfection reagent (Mirus, MIR 2306), supplemented with Opti-MEM reduced serum medium (ThermoFisher, 31985070) at 100 μl/μg of plasmids. TNF-α (Peprotech, 300-01A), IL-1β (Peprotech, 200-01B), IFN-α (Peprotech, 300-02AA) and IFN-γ (Peprotech, 300-02), were used to stimulate signaling pathways. cOmplete Protease Inhibitor Cocktail (Roche, 11697498001) and phosphatase inhibitor PhosSTOP(Roche, 4906845001) were dissolved with IP buffers. ANTI-FLAG M2 Affinity Gel (MERCK, A2220) and Monoclonal Anti-HA-Agarose (MERCK, A2095) were used in immunoprecipitation. Strep-TactinXT 4Flow resin (iba, 2-5010-002) was used in the TAP-tagged protein pull-down assay.

METHOD DETAILS

Virus infection and titration

U2OS cells inducibly expressing EYFP or HDAC5-FLAG were mock induced or induced with 100 ng/mL dox overnight. Then the cells were infected with RPXV, CPXV-BR, CPXV-E and CMLV at 0.01 pfu per cell. CPXV-BR and RPXV titration were collected at 2 days post infection, CPXV-E and CMLV was at 3 days post infection. Poxvirus containing samples were prepared with three freeze-thaw cycles and followed by sonication to release the virus particle from cell debris. BSC-1 cells were seeded in 6-well plates at 1.5 x 10^6 cells/well 24 h before titration. Viral samples were diluted to infect BSC-1 cells for 1 h with constant agitation, the inoculum was then removed and replaced with semisolid medium (DMEM supplemented with 10% FBS, 50 μg/mL of P/S, 1% carboxymethylcellulose (Sigma-Aldrich, C5678)). Two days after infection, the semisolid medium was removed, the infected cells were washed with PBS twice and fixed with PFA and stained with toluidine blue.

Viral DNA preparation

RK13 cells were infected with VACV, RPXV, MPXV, CPXV-E or CMLV at 0.001 pfu per cell. The VACV and RPXV-infected cells were collected at 2 days p.i., 3 days for MPXV and 5 days for CPXV-E and CMLV. The infected cells were prepared with three freeze-thaw cycles before sonication to maximise the release of virus particles. Samples containing viral DNA were prepared with proteinase K digestion at 56°C for 15 min, followed with heat inactivation at 95°C for 10 min.

Lentivirus preparation and transduction

HEK293T cells (3 x 10^6) in 10-cm dishes were transfected with 4 μg of pLKO.dCMV.TetO.TetR.EYFP or pLKO.dCMV.TetO.TetR.HDAC5-FLAG, 3 μg of each pCMV.dR8.91 and pMD-G plasmids. The transfection mixtures were removed after 3 h of transfection and replaced with DMEM supplemented with 30% FBS and 50 μg/mL P/S. One day post transfection, the lentivirus containing cell culture was collected, filtered with 0.45 μm filter, and supplemented with 2 μg/mL Polybrene (Sigma-Aldrich, H9268) to infect U2OS cells. The transfected cells were cultured with fresh DMEM containing 30% FBS and 50 μg/mL P/S to produce lentivirus for another lentivirus infection. After another two round lentivirus infection, transduced U2OS cells were selected with 1 μg/mL puromycin for 10 days. The resulting cell lines were routinely cultured with DMEM with 10% FBS and supplemented with 1 μg/mL puromycin.

T-REx 293 cell line construction

pcDNA4 vectors were digested with PvuI to linearize circular plasmid DNA. After DNA purification with QIAquick PCR Purification Kit (QIAGEN, 28104), 1.0 μg of the digested plasmids were transfected into 2 x 10^6 HEK.T-REx cells using TransIT-LT1 (Mirus, MIR2300). In the following day, the transfected cells were selected with medium supplemented with 5 μg/mL blasticidin (Thermo, R21001) and 100 μg/mL zeocin (InvoGen, ant-zn-1).

Immunofluorescence

One million U2OS.TetO.TetR.HDAC5-FLAG cells were grown on glass coverslips (Fisherscientific, 12313138) in six-well plates. The cells were induced with 100 ng/mL doxycycline (Melford, D43020) overnight before transfection with 0.5 μg of pcDNA3.HAcoC6, pcDNA3.HAcoC6_F72R, pcDNA3.HAcoC6_F75R or pcDNA3.HA-N1. One day after transfection, the U2OS cells were washed twice with PBS and fixed in 4% (v/v) paraformaldehyde (15710-S, Electron Microscopy Sciences) for 10 min. Following quenching with 150 mM ammonium chloride for 10 min, the fixed cells were permeabilised with 0.1% (v/v) NP-40 in PBS for 10 min. The permeabilised cells were washed twice with PBS supplemented with 1% FBS and blocked with the same washing buffer for 30 min at room temperature. The cells were then stained with Rab anti-FLAG (1:500) and Ms anti-HA (1:500) for 2 h, washed three times with the washing buffer before AlexaFluor fluorophore-conjugated secondary antibody incubation. After 1 h secondary antibody staining, the cells were washed twice with PBS and twice with ddH_2O, then mounted onto glass slides with Mowiol 4–88 (Calbiochem) supplemented 0.5 μg/mL DAPI (4′,6-diamidino2-phenylindole, Biotium). Immunofluorescence images were acquired with an LSM 700 confocal microscope (ZEISS) and processed with ZEN system software (ZEISS).

Reporter gene assay

HEK293T, HeLa or derivative HDAC5$^{-/-}$ cells were seeded in 96-well plates at 3 x 10^4 cells per well. After overnight incubation, the cells were transfected with 100 ng ISRE-Luc, GAS-Luc, NF-κB-Luc or ISG56.1-Luc plasmids and together with 10 ng of TK-Renilla. The next day, transfected cells were stimulated with 1000 unit/mL IFN-α (ISRE-Luc), 250 ng/mL IFN-γ (GAS-Luc), 10 ng/mL TNF-α (NF-κB-Luc), 100 μg/mL IL-1β (NF-κB-Luc) or infected with 40 HAU/mL SeV (ISG56.1-Luc). The IFN-α, IFN-γ, TNF-α or IL-1β stimulation was maintained for 6 h, and the SeV infection was left overnight. Supernatants of the stimulated cells were removed and the cells were lysed in 50 μL passive lysis buffer (Promega, E1910). Analysis of luciferase activity was performed using 8 μL cell lysate mixed with 50 μL renilla luciferase reagent (2 μg/mL coelenterazine in PBS) (Nanolight Technology, 350-10), or 50 μL firefly luciferase reagent [20 mM tricine, 2.67 mM MgSO4•7H2O, 0.1 mM EDTA, 33.3 mM DTT, 530 μM ATP, 270 μM acetyl-CoA, 132 μg/mL luciferin (Prolume), 5 mM NaOH, 0.26 mM MgCO3Mg(OH)2•5H2O]. The luminescence activity of the cell lysates was measured by microplate reader (BMG Labtech). Firefly luciferase value was relative to renilla luciferase control and fold induction was calculated relative to unstimulated control of each condition. FLAG-tagged RIG-I-CARD (5 ng/well), MAVS (20 ng/well), TRIFΔRIP (20 ng/well) or IRF3-5D (2.5 ng/well) were co-transfected with the above reporter plasmids to activate the ISG56 promoter driven firefly luciferase expression. Reporter gene assays were performed in triplicate and conducted at least three times.

Affinity purification

HEK293T cells were seeded at 10-cm dishes at 3 x 10^6 cells per plate. In the next day, cells were transfected with plasmids expressing indicated proteins. Two days post transfection, the transfected cells were collected and lysed with an IP buffer (200 mM NaCl, 50 mM Tris, pH 7.0, supplemented with protease inhibitor, cOmplete, Merck) at 4°C with constant agitation for 2 h. Cell lysates were precleared by centrifugation at 13,000 rpm, 4°C, 30 min. Supernatants were collected and FLAG-tagged or HA-tagged proteins were immunoprecipitated with anti-FLAG or anti-HA antibody conjugated affinity agarose respectively. TAP-tagged proteins were pulled-down with Strep-Tactin resin. The IP or pull-down of tagged proteins were performed at 4°C with constant agitation for at least 2 h. Input and IP samples were resolved on SDS-polyacrylamide gels and then transferred to nitrocellulose membranes. Proteins of interest were probed with antibodies as indicated.

Recombinant protein expression and purification

N-terminally GST-tagged C6 was expressed in *E. coli* BL21(DE3)pLysS cells (Promega). Cells were grown at 37°C in 2xTY medium (Sigma-Aldrich) supplemented with 50 μg/L ampicillin and 12.5 μg/L chloramphenicol with constant agitation at 200 rpm until OD_{600} was between 0.6 and 0.9. Protein expression was induced with 0.4 mM isopropyl β-D-thiogalactopyranoside (IPTG) for 4 h at 37°C before cells were harvested by centrifugation at 4,000 *g* in Beckman Avanti J-HC Refrigerated Centrifuge (Beckman Coulter) for 20 min. Cell pellets were collected and stored at – 70°C for later use. Thawed cells were resuspended in lysis buffer at 4°C containing 20 mM Tris pH 7.5, 300 mM NaCl, 1 mM dithiothreitol (DTT), 0.5 mM $MgCl_2$, 0.05% Tween 20, 1 mM β-mercaptoethanol supplemented with 200 unit DNAse I (Thermo Scientific) per liter of cell pellets and 1 tablet of cOmplete EDTA-free protease inhibitor cocktail (Merck) and lysed by passing twice through a TS series cell disruptor (Constant Systems) at 24 kpsi. Cell lysates were subjected to centrifugation at 40,000 *g* and the supernatant was incubated at 4°C for 2 h with glutathione Sepharose 4B beads (Cytiva) pre-equilibrated with washing buffer containing 20 mM Tris pH 7.5, 300 mM NaCl, 1 mM DTT to allow GST-C6 to be captured on beads. After incubation, the beads were washed twice using the washing buffer before elution by incubating with 25 mM reduced glutathione (GSH, Sigma Aldrich) in 20 mM Tris pH 7.5, 300 mM NaCl, 1 mM DTT at 4°C for 1 h. The eluent was buffer exchanged into 20 mM Tris pH 7.5, 200 mM NaCl using a HiPrep 26/60 desalting column (Cytiva) and concentrated to 2 mg/mL before diluting with 100% glycerol to a final concentration of 1 mg/mL in 50% glycerol. The 50% glycerol stock of GST-C6 was stored at 4°C for use in GST pull-down studies.

In vitro expression system

Cell free protein expression system TNT SP6 High-Yield Wheat Germ Protein Expression System was purchased from Promega, L3260. pF3A.TAPcoC6, pF3A.N1-TAP, pF3A.HDAC5-HA, pF3A.HDAC5N-HA HDAC1-HA, pF3A.m.yc-HDAC4, pF3A.m.yc-H4N, pF3A.m.yc-HDAC5, pF3A.m.yc-H5N, pF3A.m.yc-H5NΔ1, pF3A.m.yc-H5NΔ2, pF3A.m.yc-H5NΔ3, pF3A.m.yc-H4N, pF3A.m.yc-H4NΔ1, pF3A.m.yc-H4NΔ2 and pF3A.m.yc-H4NΔ3 were used for *in vitro* transfection/translation to express proteins of interest following the manufacturer's protocol. Tagged proteins were immunoprecipitated with affinity resin, washed 3 times with IP buffer (Buffer for Anti-HA co-immunoprecipitation contains 150 mM NaCl, 50 mM Tris-HCl, 1% NP40, pH 7.0; Buffer for Streptavidin co-precipitation is Phosphate-buffered saline supplemented with 0.5% NP-40) and then analyzed by SDS-PAGE and immunoblotting.

GSH beads pull-down of GST-C6 and GST

Purified GST and GST-tagged proteins (for purification, see recombinant protein expression and purification above) were captured onto magnetic glutathione beads as the bait proteins by incubating pre-washed beads with approximately 0.5 nmol of the proteins diluted in 200 μL of the washing buffer (20 mM HEPES pH 7.5, 200 mM NaCl, 1 mM EDTA, 1 mM DTT, 0.1% v/v NP-40) with shaking at room temperature for 10 min. The beads were collected via a magnetic plate and washed three times with 200 μL of the washing buffer before subjected to pull-down experiments. Human cDNAs of full-length and truncated HDAC4 and HDAC5 were subcloned into pF3A WG (BYDV) vector (Promega) and incubated with the Wheat Germ (WG) Cell Free Expression mix (Promega) following the manufacturer's protocol at 25°C for 2 h to induce protein expression. The final reaction mixtures would contain 30 μL of WG mix and approximately 3 μg of expression plasmids dissolved in 20 μL DNAse free ddH_2O. After reactions were complete, each mixture was diluted with 160 μL of the washing buffer to a total of 210 μL. Half of each diluted reaction mixture was incubated with the magnetic glutathione beads bound to GST-tagged C6 proteins, while the remaining half of the mixture was incubated with beads bound to GST alone as negative controls. After 60 to 90 min incubation at room temperature with constant agitation, the beads were pulled down and washed three times with 200 μL of the washing buffer. The bait-prey mixtures were finally eluted by incubating the beads with 50 μL of the elution buffer (20 mM HEPES pH 7.5, 200 mM NaCl, 1 mM EDTA, 1 mM DTT, 0.1% v/v NP-40, 25 mM reduced glutathione) at room temperature for 15 min. The eluents were collected and analyzed by SDS-PAGE and immunoblotting.

Prediction of the C6-HDAC5 co-structure by AlphaFold2

Predictions of C6-HDAC5 co-structure were performed using the sequence of VACV C6 with full-length or N-terminal 650 aa of HDAC5 sequence as an input and all default parameters via AlphaFold2-multimer. The models obtained have been deposited with this manuscript.

QUANTIFICATION AND STATISTICAL ANALYSIS

The value of n refers to the number of biological replicates, and is indicated in the respective figure legends. Data from Figure S1B was analyzed using one-way Welch's ANOVA test, performed with the statistics module from GraphPad PRISM. Data from Figure S4B was analyzed using two-way Welch's ANOVA test. The remaining statistical analysis was carried out using two-tailed t tests. Statistical significance is expressed as follows: not significant (ns), *$p < 0.05$, **$p < 0.01$, ***$p < 0.001$, ****$p < 0.0001$. All data points represent the mean ± SEM.

Cell Reports, Volume *43*

Supplemental information

HDAC5 enhances IRF3 activation and is targeted for degradation by protein C6 from orthopoxviruses including *Monkeypox virus* and *Variola virus*

Yongxu Lu (陆泳旭), Yiqi Zhao (赵奕祺), Chen Gao (高晨), Shreehari Suresh, Jinghao Men (门靖浩), Amelia Sawyers, and Geoffrey L. Smith

S 1

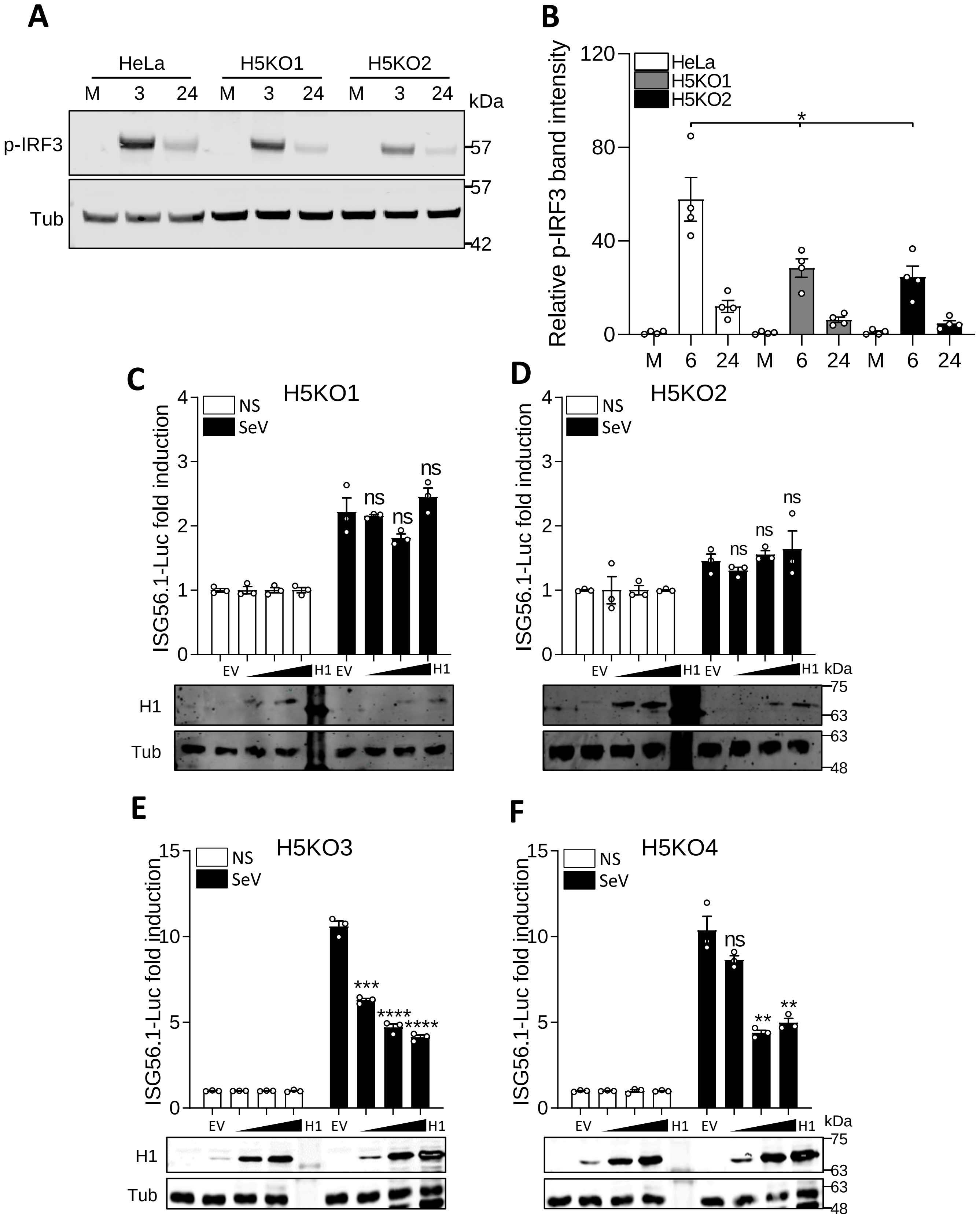
A
HeLa
H5KO1
H5KO2
M
3
24
kDa
p-IRF3
Tub
57
42
B
Relative p-IRF3 band intensity
HeLa
H5KO1
H5KO2
*
0
40
80
120
M
6
24
C
H5KO1
D
H5KO2
NS
SeV
ISG56.1-Luc fold induction
ns
EV
H1
H1
Tub
75
63
48
E
H5KO3
F
H5KO4
***
****
**

Fig. S1. IRF3 activation is attenuated in HDAC5$^{-/-}$ cells and cannot be rescued by HDAC1 or 4 over-expression. (Related to Fig. 1)

(A). Immunoblot showing p-IRF3 levels in parental HeLa and two HDAC5$^{-/-}$ cells. HeLa, H5KO1 and H5KO2 were infected with SeV, collected at different times p.i., and analysed by immunoblotting for levels of phosphorylated IRF3 (p-IRF3) and α-tubulin.

(B). The p-IRF3 level in (A) was calculated relative to α-tubulin (Tub) ± s.e.m. from 4 independent experiments. Data was analysed using one-way Welch's ANOVA test.

(C-F). Expression of HDAC1 does not rescue enhanced IRF3 pathway activation in HDAC5$^{-/-}$ cells. HDAC5$^{-/-}$ cells derived from HeLa (H5KO1 and H5KO2) or HEK293T (H5KO3 and H5KO4) cells were transfected with ISG56.1-Luc, TK-*Renilla* and increasing doses of plasmids expressing FLAG-tagged HDAC1 overnight. Transfected cells were then infected with SeV overnight to activate IRF3 pathway. The cells were then collected and firefly luciferase was measured and normalised to renilla luciferase control. The fold induction was calculated relative to unstimulated controls. Each condition was performed in triplicate. For each reporter gene assay, protein extracts were prepared and analysed by immunoblotting for FLAG-tagged HDAC1 and α-tubulin. The positions of molecular mass markers in kDa are indicated on the right.

Data are presented as mean ± s.e.m., n = 3 independent experiments. ns = not significant, *$p < 0.05$, **$p < 0.01$, ***$p < 0.001$, ****$p < 0.0001$.

S 2

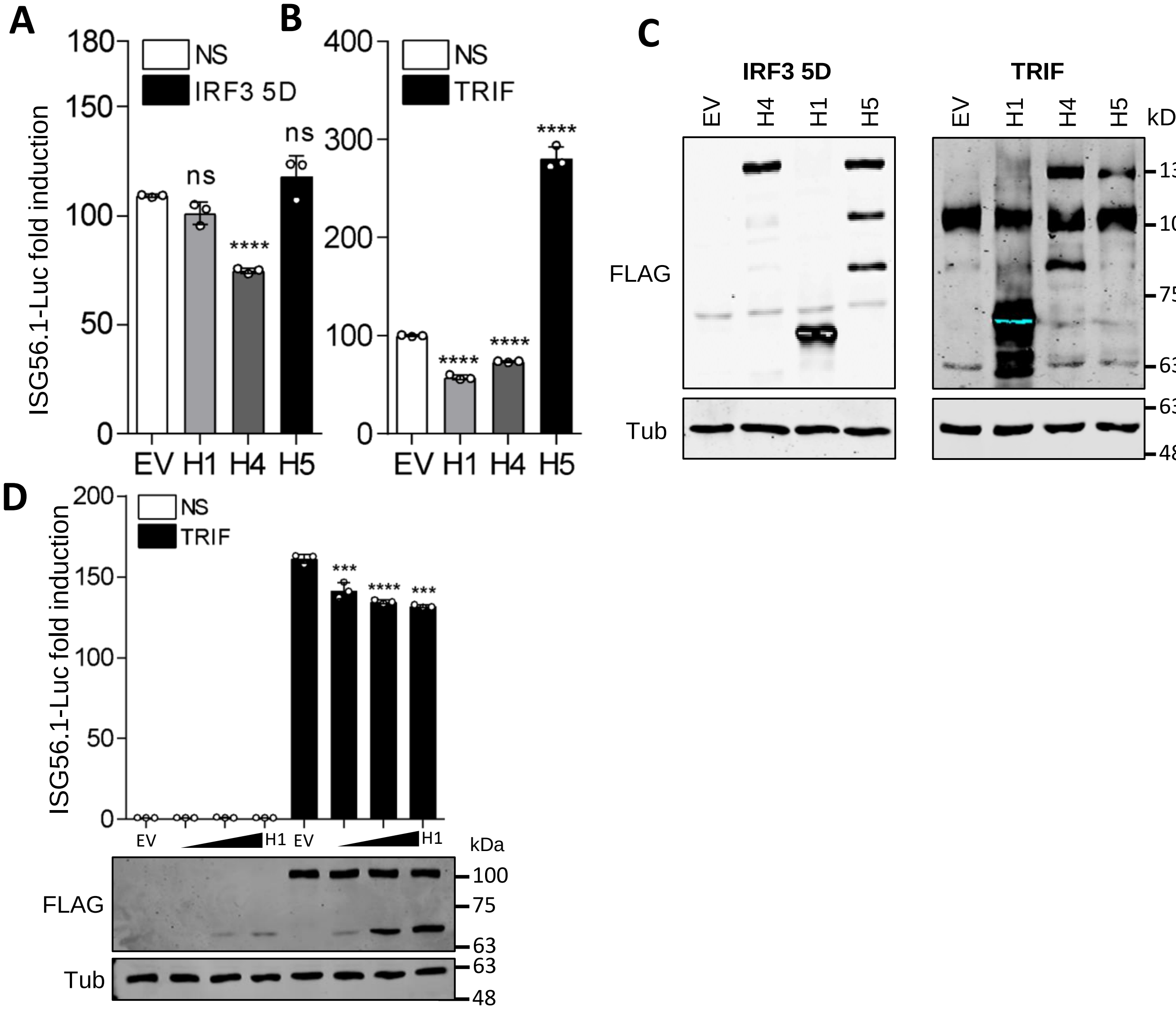

Fig. S2. HDAC5 enhances TRIF-mediated IRF3 activation. (Related to Fig. 3)

(A). HDAC5 does not enhance phospho-IRF-3–mediated IRF3 pathway activation. HEK293T cells seeded in 96-well plates were co-transfected with 100 ng ISG56.1-Luc, 10 ng TK-*Renilla*, 100 ng FLAG-tagged HDAC1, HDAC4, HDAC5 or empty vector (EV), and 5 ng of a mutant IRF3 (IRF3-5D). At 18 h post transfection, the cell lysates were collected and the firefly luciferase activity was measured and normalised to renilla luciferase as described in Fig. 1. Protein extracts were also used for immunoblotting as shown in (C).

(B). HDAC5, but not HDAC1 or HDAC4, enhances TRIF-mediated IRF3 activation in HEK293T cells. As in (A), except that 20 ng of TAP-tagged TRIFΔRIP was co-transfected to activate the IRF3 pathway for18 h. Cell lysates were then prepared and used to measure luciferase activity and for immunoblotting for expression of the FLAG-tagged proteins as shown in (C).

(C). Immunoblots for FLAG-tagged proteins and α-tubulin in cell lysates from (A) and (B).

(D). Expression of HDAC1 does not rescue TRIF-mediated IRF3 pathway activation in HDAC5$^{-/-}$ cells. H5KO3 cells were transfected with ISG56.1-Luc, TK-*Renilla*, TAP-TRIFΔRIP and HDAC1-FLAG expressing plasmids overnight. The HDAC1-FLAG expressing plasmid was transfected at 0, 10, 50, or 100 ng per well. The relative ISG56.1-Luc reporter expression was analysed as in Fig. 1. Bottom panels show immunoblots for HDAC1-FLAG, TAP-TRIFΔRIP and α-tubulin. In the immunoblots shown in (C) and (D), the positions of molecular mass markers in kDa are shown in the right.

Data are presented as mean ± s.e.m., n ≥ 3 independent experiments. ns = not significant, ***$p < 0.001$, ****$p < 0.0001$.

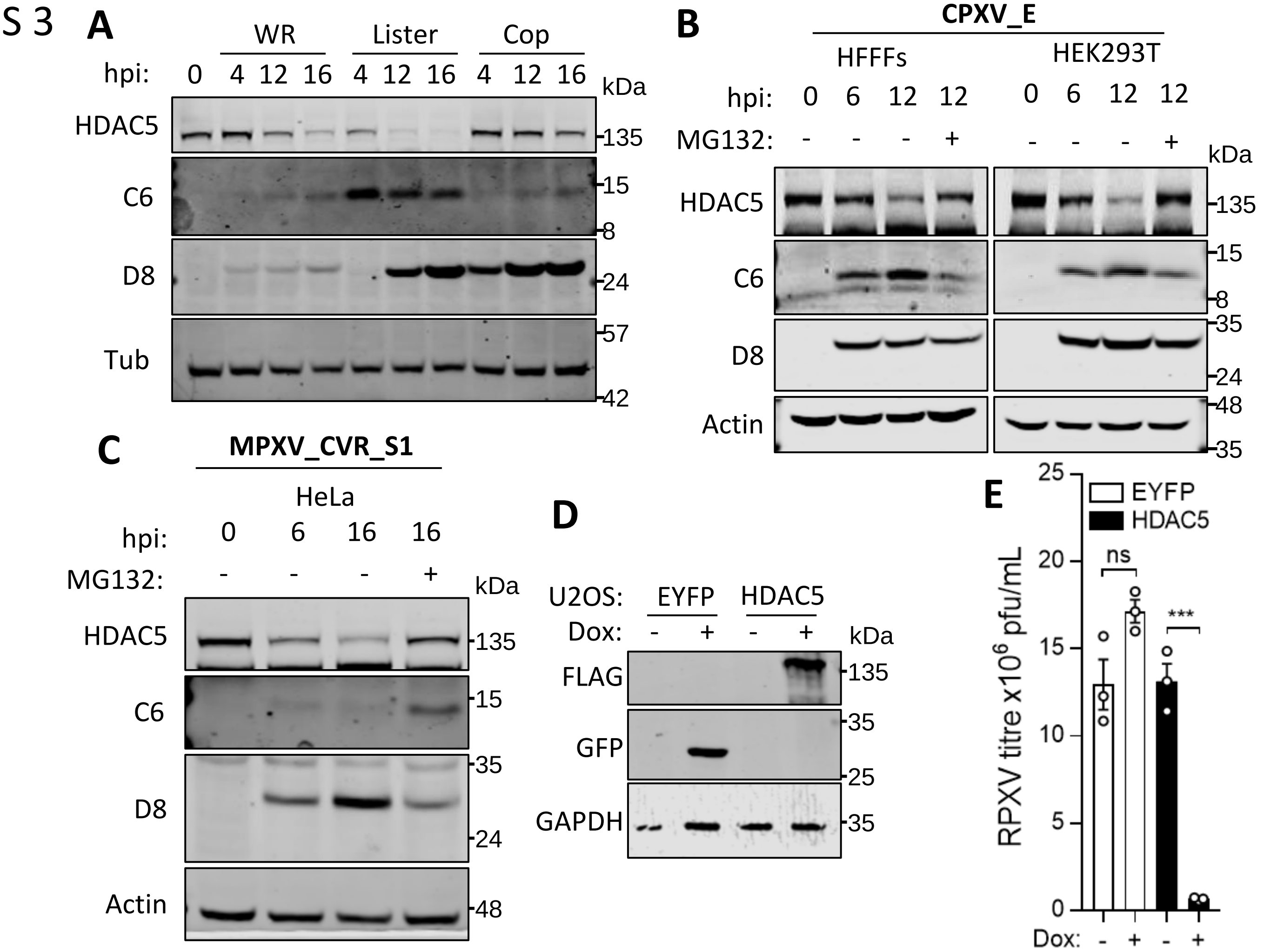

Fig. S3. HDAC5 restricts RPXV replication and is targeted for degradation by CPXV_E, MPXV_CVR_S1 and several VACV strains. (Related to Fig. 4)

(A). VACV strains WR, Copenhagen and Lister induce HDAC5 degradation during infection.

HFFFs were infected with VACV strains WR, Lister or Copenhagen (Cop) at 5 pfu per cell for 2 h and then the inoculum was replaced with fresh medium and incubated for the indicated times. Cell lysates were prepared and analysed by immunoblotting with the indicated antibodies.

(B). CPXV-E induces HDAC5 degradation during infection. As in (Fig. 4A), HFFFs or HEK293T cells were infected with CPXV-E at 5 pfu per cell for 2 h and then the inoculum was replaced with fresh medium supplemented with 10 µM MG132 (+) or equal volume of the carrier DMSO (-) and incubated for the indicated times. Cell lysates were prepared and analysed by immunoblotting with the indicated antibodies.

(C). As in (Fig. 4D). HeLa cells were infected with MPXV_CVR_S1 at 5 pfu per cell for 2 h and then the inoculum was replaced with fresh medium supplemented with 10 µM MG132 (+) or equal volume of the carrier DMSO (-) and incubated for the indicated times. Cell lysates were prepared and analysed by immunoblotting with the indicated antibodies.

(D). U2OS cells inducibly-expressing EYFP or HDAC5-FLAG. U2OS.TetR.EYFP or U2OS.TetR.HDAC5-FLAG cells were mock-induced (-) or induced with 100 ng/mL dox (+) for 18 h, and then cell lysates were prepared and analysed by immunoblotting. In (A-D), the positions of molecular mass markers are indicated in kDa on the right.

(E). Expression of HDAC5 in U2OS cells restricts RPXV replication. As in Fig. 4G. U2OS.TetR.EYFP or U2OS.TetR.HDAC5-FLAG cells were mock-induced (-) or induced with 100 ng/mL dox for 18 h (+) and then infected with RPXV at 0.01 pfu per cell for 2 d. The infectious virus present in the culture supernatant and infected cells was determined by plaque assay on BSC-1 cells.

Data are presented as mean ± s.e.m., n ≥ 3 independent experiments. ns = not significant, ***$p < 0.001$.

S 4

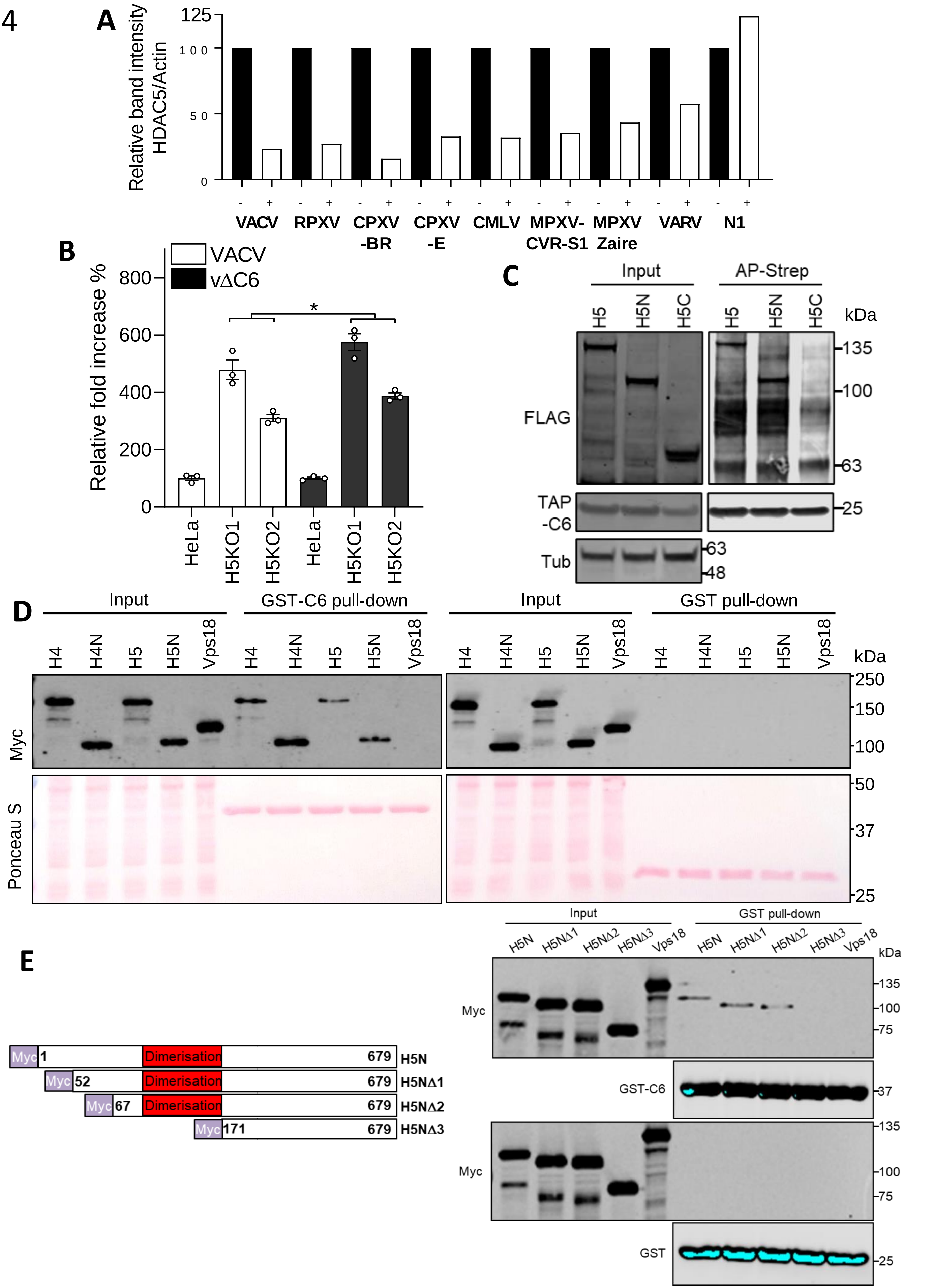
A
Relative band intensity HDAC5/Actin
125
100
50
0
VACV
RPXV
CPXV -BR
CPXV -E
CMLV
MPXV-CVR-S1
MPXV Zaire
VARV
N1
B
VACV
vΔC6
Relative fold increase %
800
600
400
200
0
HeLa
H5KO1
H5KO2
C
Input
AP-Strep
H5
H5N
H5C
kDa
FLAG
TAP -C6
Tub
135
100
63
25
48
D
Input
GST-C6 pull-down
GST pull-down
H4
H4N
Vps18
Myc
Ponceau S
250
150
50
37
E
Myc
Dimerisation
H5NΔ1
H5NΔ2
H5NΔ3
1
52
67
171
679
GST-C6
GST
75

Fig. S4. C6 interacts directly with the N-terminal region of HDAC4 and HDAC5 leading to their degradation. (Related to Fig. 5).

(A). Relative HDAC5 abundance in cells expressing (+) or not expressing (-) C6 proteins from different orthopoxviruses. The graph shows the band intensity of HDAC5 from Fig. 5D normalised to actin and comparing the mock-induced (-, 100%) to dox induced (+) samples.

(B). Deletion of C6 enhances the difference in viral yield between HeLa and HDAC5$^{-/-}$ cells. Parental HeLa, H5KO1 and H5KO2 cells were infected with vΔC6 or wt VACV at MOI=0.01. Two d p.i., the supernatant and infected cells were collected and infectious virus was titrated by plaque assay on BSC-1 cells. Data was analysed using two-way Welch's ANOVA test. n = 3 independent experiments. $^{*}p < 0.05$

(C). C6 co-precipitates with the HDAC5 N-terminal region. HEK293T cells were co-transfected with plasmids encoding TAP-C6 and FLAG-tagged full length HDAC5, N-terminal HDAC5 (H5N, aa 1-679) or C-terminal HDAC5 (H5C, aa 680-1122). Cell lysates were prepared and TAP-C6 was AP by Strep-Tactin beads. Inputs and AP proteins were analysed as described in (Fig. 5).

(D) HDAC5 and HDAC4 bind directly to C6. The indicated myc-tagged HDAC4 (H4), N-terminal HDAC4 (H4N, aa 1-650), HDAC5 (H5), H5N or Vsp18 were expressed *in vitro* using the wheat germ cell-free transcription and translation system. The *in vitro* expressed proteins were AP with glutathione beads bound to glutathione S-transferase (GST)-tagged C6 (middle panel) or GST (bottom panel). The AP proteins were analysed by immunoblotting using anti-myc antibody and the GST-C6 and GST were stained with ponceau S.

(E). More detailed mapping of the HDAC5 region required for binding C6. Same as in (D) The indicated myc-tagged HDAC5 mutants (top panel) or Vsp18 were expressed *in vitro* and AP with glutathione beads bound to glutathione S-transferase (GST)-tagged C6 (middle panel) or GST (bottom panel). The AP proteins were analysed by immunoblotting using anti-myc or anti-GST antibodies. In (C-E) the positions of molecular mass markers are shown in kDa on the right.

S 5

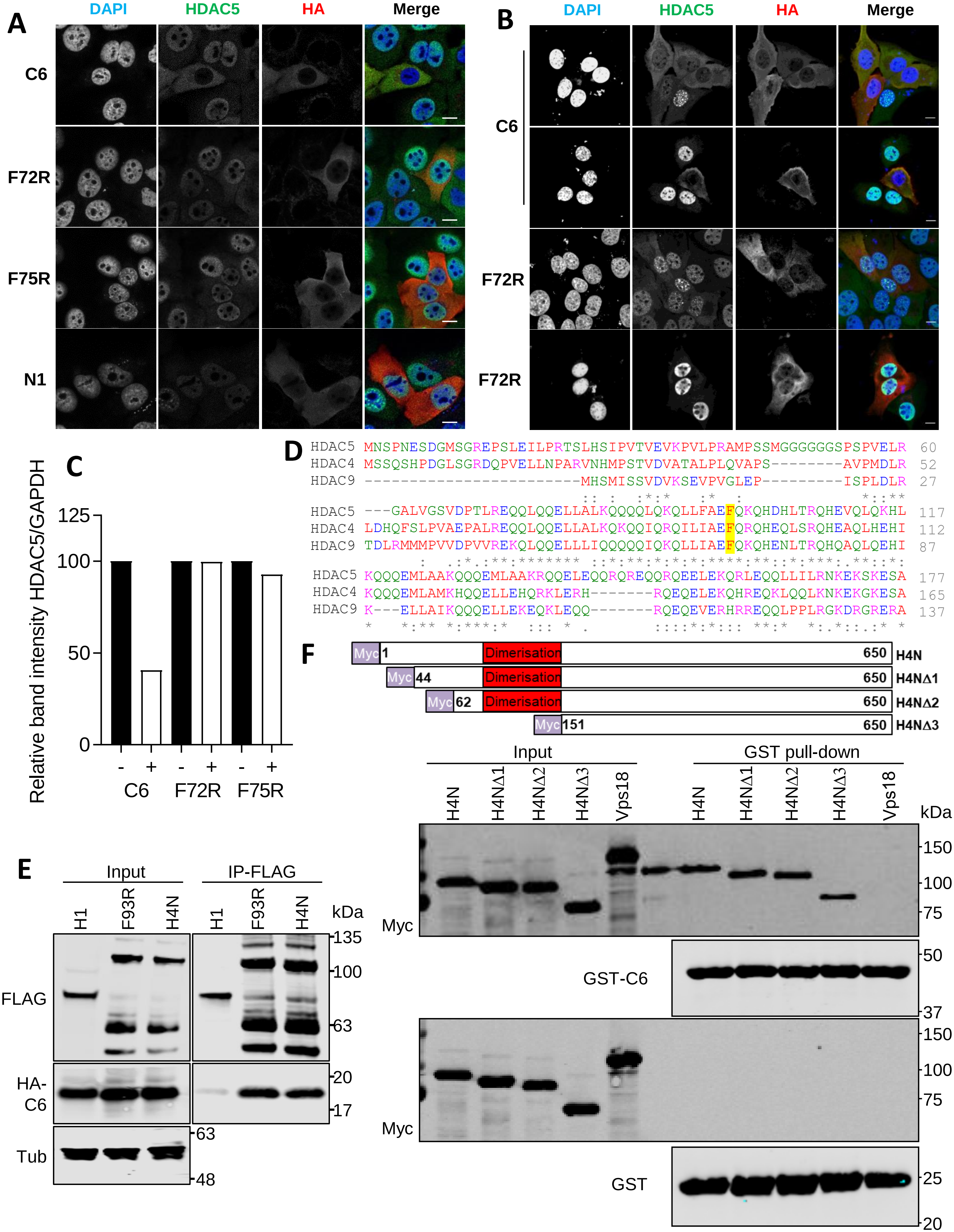
A
DAPI
HDAC5
HA
Merge
C6
F72R
F75R
N1
B
DAPI
HDAC5
HA
Merge
C6
F72R
F72R
C
Relative band intensity HDAC5/GAPDH
125
100
50
0
- + - + - +
C6 F72R F75R
D
HDAC5 MNSPNESDGMSGREPSLEILPRTSLHSIPVTVEVKPVLPRAMPSSMGGGGGGSPSPVELR 60
HDAC4 MSSQSHPDGLSGRDQPVELLNPARVNHMPSTVDVATALPLQVAPS--------AVPMDLR 52
HDAC9 ----------------------MHSMISSVDVKSEVPVGLEP---------ISPLDLR 27
HDAC5 ---GALVGSVDPTLREQQLQQELLALKQQQQLQKQLLFAEFQKQHDHLTRQHEVQLQKHL 117
HDAC4 LDHQFSLPVAEPALREQQLQQELLALKQKQQIQRQILIAEFQRQHEQLSRQHEAQLHEHI 112
HDAC9 TDLRMMMPVVDPVVREKQLQQELLLIQQQQQIQKQLLIAEFQKQHENLTRQHQAQLQEHI 87
HDAC5 KQQQEMLAAKQQQEMLAAKRQQELEQQRQREQQRQEELEKQRLEQQLLILRNKEKSKESA 177
HDAC4 KQQQEMLAMKHQQELLEHQRKLERH-------RQEQELEKQHREQKLQQLKNKEKGKESA 165
HDAC9 K---ELLAIKQQQELLEKEQKLEQQ-------RQEQEVERHRREQQLPPLRGKDRGRERA 137
E
Input
IP-FLAG
H1
F93R
H4N
H1
F93R
H4N
kDa
135
100
63
20
17
63
48
FLAG
HA-C6
Tub
F
Myc 1 Dimerisation 650 H4N
Myc 44 Dimerisation 650 H4NΔ1
Myc 62 Dimerisation 650 H4NΔ2
Myc 151 650 H4NΔ3
Input
GST pull-down
H4N
H4NΔ1
H4NΔ2
H4NΔ3
Vps18
H4N
H4NΔ1
H4NΔ2
H4NΔ3
Vps18
kDa
150
100
75
50
37
150
100
75
25
20
Myc
GST-C6
Myc
GST

Fig. S5. C6 induces HDAC5 re-localisation and interacts directly with multiple binding sites in the HDAC4 N-terminal region (Related to Figs. 6 & 7).

(A). HDAC5 re-localisation to the cytoplasm is induced by C6, but not C6 mutants F72R or F75R. U2OS.HDAC5 cells were transfected with 0.5 µg of plasmids expressing HA-tagged N1, C6 or C6 mutants F72R or F75R for 24 h. Cells were induced with 100 ng/mL dox to express HDAC5-FLAG overnight. Cells were then fixed and immunostained with anti-FLAG and anti-HA antibodies, and DNA was stained with DAPI. Cell images were obtained via confocal microscopy. Scale bar, 20 µm.

(B). Independent immunofluorescence images showing that C6, but not C6 mutants F72R and F75R, induced HDAC5 re-localisation. Scale bar, 20 µm.

(C). Relative HDAC5 abundance in cells expressing (+) or not expressing (-) C6 or C6 mutants F72R and F75R. The graph shows the band intensity of HDAC5 from Fig. 6G normalised to GAPDH and comparing the mock-induced (-, 100%) to dox induced (+) samples.

(D). Amino acid sequence alignment of HDAC5, HDAC4 and HDAC9. The position of HDAC5 F98 and the corresponding aa in HDAC4 and HDAC9 are highlighted in yellow.

(E). HDAC4 F93 is not essential for HDAC4 and C6 co-precipitation. TAP-tagged C6 and FLAG-tagged HDAC1, HDAC4-N or HDAC4-N with a F93R mutation were co-expressed in HEK293T cells by transfection and were precipitated with anti-FLAG antibody. Input (left) and purified proteins (right) were analysed by immunoblotting with the indicated antibodies.

(F). Mapping of HDAC4 domain(s) required for binding C6. As in Fig. S4C, except that myc-tagged HDAC4 mutants (top panel) were expressed *in vitro* using the wheat-germ cell free transcription-translation system and were AP with either GST-C6 (middle panel) or GST (bottom panel). In C & D, the positions of molecular mass markers are shown in kDa on the right.
